# Research progress on the antiviral activities of natural products and their derivatives: Structure–activity relationships

**DOI:** 10.3389/fchem.2022.1005360

**Published:** 2022-10-12

**Authors:** Yajing Guo, Anna Ma, Xinyan Wang, Chen Yang, Xi Chen, Gen Li, Feng Qiu

**Affiliations:** ^1^ School of Chinese Materia Medica, Tianjin University of Traditional Chinese Medicine, Tianjin, China; ^2^ School of Pharmaceutical Engineering of Traditional Chinese Medicine, Tianjin University of Traditional Chinese Medicine, Tianjin, China; ^3^ Tianjfin State Key Laboratory of Modern Chinese Medicine, Tianjin University of Traditional Chinese Medicine, Tianjin, China

**Keywords:** natural products, antiviral, derivatization, resistance, structural-activity relationship

## Abstract

Viruses spread rapidly and are well-adapted to changing environmental events. They can infect the human body readily and trigger fatal diseases. A limited number of drugs are available for specific viral diseases, which can lead to non-efficacy against viral variants and drug resistance, so drugs with broad-spectrum antiviral activity are lacking. In recent years, a steady stream of new viral diseases has emerged, which has prompted development of new antiviral drugs. Natural products could be employed to develop new antiviral drugs because of their innovative structures and broad antiviral activities. This review summarizes the progress of natural products in antiviral research and their bright performance in drug resistance issues over the past 2 decades. Moreover, it fully discusses the effect of different structural types of natural products on antiviral activity in terms of structure–activity relationships. This review could provide a foundation for the development of antiviral drugs.

## 1 Introduction

Recently, numerous viral diseases originating from wildlife hosts have posed a serious threat to the life of humans. These viruses have included the Ebola virus (Zhu et al., 2020), human immunodeficiency virus (HIV) (Yonekawa et al., 2019), and influenza A virus (IAV) (Joseph et al., 2017). Close contact between humans and domestic animals and populations of wild animals has increased the risk of virus transmission between species. The International Committee on Taxonomy of Viruses approved and promulgated the latest classification of viruses in 2021, which contains 9,110 viruses (Walker et al., 2021). The increasing number of viruses demonstrates their biological diversity and rapid adaptability, and reflects the potential harmfulness of viruses.

Viruses destroy the structure and function of host cells and cause serious damage to the host by multiplying. They also evolve at a fast rate to adapt to the host’s internal environment. For example, there were 2,682 male and 2,455 female deaths from infection by the Dengue virus (DENV) and its variants over the past 3 decades in Brazil, with symptoms of severe internal bleeding, circulatory collapse, and shock (Nunes et al., 2019). Many diseases caused by viral infections are transmissible, lethally harmful, and difficult to cure.

Vaccines and antiviral drugs are the two main strategies for fighting viruses. In general, vaccines are considered the best means for preventing viral infections. However, vaccine development requires rigorous processes, which are time-consuming. Also, the vaccination rates and outcome data are not impressive in older populations, which necessitates use of antiviral agents to complement vaccines (Demicheli et al., 2018). Only a few antiviral drugs have been developed to prolong the life of patients, but they had significant disadvantages: high price, resistance, and non-efficacy against viral variants. Coronavirus disease 2019 (COVID-19) occurs due to infection by severe acute respiratory syndrome-coronavirus 2 (SARS-CoV-2) infection. COVID-19 continues to wreak havoc on healthcare and economic systems worldwide. The number of infections and deaths due to SARS-CoV-2 keeps rising, new strains of the virus are emerging, and definite efficacious treatment is not available (Barlow et al., 2020). Existing therapeutics cannot stop infection by or transmission of viruses, and humankind cannot wait for the research and development of new antiviral drugs.

“Natural products” (NPs) are chemical substances of natural origin. They have complicated structures and a wide variety of biological activities (Newman and Cragg, 2016). Many active components of NPs and their derivatives possess antiviral activity, such as alkaloids, quinones, flavonoids, terpenoids, glycans, organic acids, and others (Supplementary Table S1). Newman et al. concluded that, in the last 28 years, the drugs developed based on NPs were 63.1% of all small-molecule drugs (Newman and Cragg, 2020). That figure demonstrates the great potential of NPs and their derivates in the development of new drugs. Wright suggested continuation of exploration of NPs as a source for drug development based on existing research. He suggested avoiding the complicated steps of synthesis “from scratch” and rationalizing application of resources for solving threats to the life and health of humans (Wright, 2019).

Up to now, to our knowledge, there is no reported data to conclude the relationships between the structure of each natural product component and its antiviral activity. This review summarized the research progress of antiviral NPs and their derivatives in the past 2 decades. We focused on the structure-activity relationships between various types of active ingredients in NPs and their antiviral activity, mainly alkaloids, quinones, flavonoids, terpenoids, glycans, organic acids and others. We also discussed the development potential of natural products in resolving drug resistance problems, and provided a rationale for in-depth development of antiviral drugs.

## 2 Alkaloids

**FIGURE 1:**
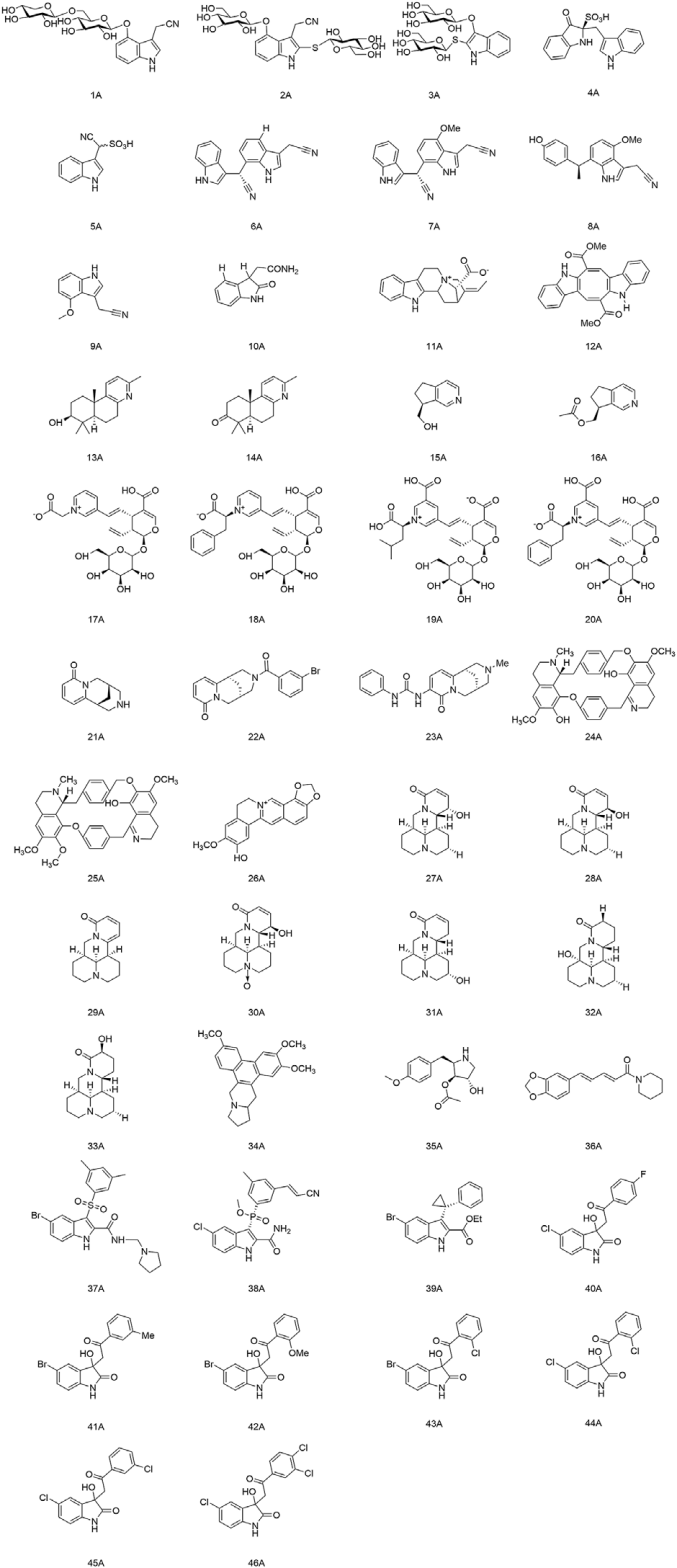
Structures of alkaloids (and their derivatives) with antiviral activity.

Alkaloids represent a structurally diverse group of nitrogen-containing bases. Most of them show significant pharmacological activities. In particular, the alkaloids with antiviral activity mainly include the following categories: indole, terpenoid, quinolinine, isoquinoline, indolizidine, quinolizidine, pyrrolidine and piperidine. The structures of alkaloids and their derivatives mentioned in this review are shown in Figure 1.

### 2.1 Indole alkaloids

Meng and colleagues discovered two new indole alkaloid diglycosides, isatigotindolediosides C (1A) and isatigotindolediosides E (2A), along with one known analog Calanthoside (3A), isolated from an aqueous extract of *Isatis indigotica* roots (Meng et al., 2017b). (1A) and (2A) showed equal inhibitory activity to the (3A) for coxsackievirus B3 (CVB3), with an IC_50_ of 33.3 µM. Also, Meng’s team separated eight additional indole alkaloid sulfonic acids from the aqueous extract of *I. indigotica* roots, including isatibisindosulfonic acid B (4A) and isatindosulfonic acid B (5A), which had activity against CVB3 and influenza virus A, respectively (Meng et al., 2017a). Chen’s team identified seventeen alkaloids from the aqueous extract of *I. indigotica* roots. Compounds (6A), (7A), (8A), and (9A) had activity against influenza viruses, and (10A) inhibited CVB3 replication with an IC_50_ value of 6.87 μM (Chen et al., 2012). Moradi and his team discovered that the total alkaloids of an extract of *Peganum harmala* seeds had a highly inhibitory effect upon IAV replication in Madin-Darby canine kidney (MDCK) cells. They could restrain the RNA replication and polymerase activity of the IAV without affecting its hemagglutination inhibition and virucidal activity, so they could be developed as agents against the IAV (Moradi et al., 2017). Zhang and his colleagues isolated a novel indole alkaloid, 17-nor-excelsinidine (11A), from *Alstonia scholaris* and it was significantly more potent than acyclovir against the herpes simplex virus (HSV) and adenoviruses, with an EC_50_ of 1.09 and 0.94 μg/ml, respectively (Zhang et al., 2014). Esteves and his team isolated caulerpin (12A) from the marine green alga *Caulerpa racemose*, and showed anti-Chikungunya virus (CHIKV) activity, and its derivatives were promising as anti-CHIKV drugs (Esteves et al., 2019). Macedo and coworkers revealed that (12A) can inhibit the alpha and beta phases of the replication cycle of the herpes zoster type-1 virus, as well as could be a substitute for acyclovir (Macedo et al., 2012).

### 2.2 Terpenoid alkaloids

Li and his team isolated two diterpenoid forsyqinlingines (13A), (14A) and two C9-monoterpenoid alkaloids (15A), (16A) from *Forsythia suspensa*, all of them showed antiviral effects against the IAV and respiratory syncytial virus *in vitro* (Li W. et al., 2021; Li et al., 2022). Yu and his collaborates separated and identified nine new alkaloids from the aqueous extract of *Lonicera japonica* flower buds. Compounds (17A), (18A), (19A), and (20A) demonstrated activity against influenza viruses, and (18A) inhibited replication of coxsackieviruses (Yu et al., 2013).

### 2.3 Quinolinine and isoquinolinine alkaloids

(−)-Cytisine (21A) is a quinoline alkaloid with antiviral activity. It is mainly isolated from plants of the Leguminosae family (Gotti and Clementi, 2021). The structural modifications of (21A) have focused on its secondary nitrogen atom and 2-pyridone core. Tsypysheva and collaborators revealed that derivative (22A) with introduction of *m*-bromobenzamide on the secondary nitrogen atom and (23A) with an aryl-substituted urea moiety on the 2-pyridone core could improve the anti-influenza-virus activity of (21A) (ED_50_ = 109 μg/ml) with ED_50_ values of 44 and 57 μg/ml, respectively. They provided a reference for further targeting and optimizing of the antiviral activity of quinoline alkaloids (Tsypysheva et al., 2013). In addition, they discovered that (−)-cytisine derivatives have activity against DENV-2. The attachment and entry of E proteins targeting the DENV could be inhibited by introduction of a substituted thioamide or thiocarbamide fragment at the 3-position of the 2-pyridone core, as well as insertion of a fragment that formed a donor–acceptor bond (Tsypysheva et al., 2021). Silva and colleagues extracted a bisbenzylisoquinoline alkaloid, warifteine (24A), from the rhizomes of *Cissampelos sympodialis*, which proved to be an anti-DENV (da Silva et al., 2021a). Subsequently, they found that (24A) and methylwarifteine (25A) had strong effects against the Zika virus *in vitro*, and could be used as a pharmacophore or lead compounds to counteract Zika-virus infection (da Silva et al., 2021b). Zeng’s team identified that dehydrocheilanthifoline (26A) had anti-hepatitis B virus (HBV) activity *in vitro*, making it a promising drug candidate for the treatment of HBV infection (Zeng et al., 2013).

### 2.4 Indolizidine and quinolizidine alkaloids

Pan’s team discovered that several bitter ginseng alkaloids, such as compounds (27A), (28A), and (29A) inhibited replication of influenza viruses, whereas compounds (30A), (31A), (32A), and (33A) showed activity against CVB3 (Pan et al., 2015). Xi and colleagues suggested that Tylophorine B (34A) had high affinity for the RNA of the tobacco mosaic virus (TMV) and the starting point of its oriRNA assembly, with an IC_50_ of 2.4 nM against TMV RNA. Presumably, (34A) contributed to the viral-suppressive effect by binding to oriRNA and interfering with viral assembly (Xi et al., 2006).

### 2.5 Pyrrolidine alkaloids and piperidine alkaloids

Quintana and collaborators demonstrated that anisomycin (35A) (derived from *Botrytis cinerea*) had activity against the DENV and Zika virus by inhibiting viral replication (Quintana et al., 2020). Huang et al. discovered significant inhibition of SARS-CoV-2 replication in Vero E6 cells at the nanomolar level with relatively non-toxic concentrations of (35A) (Huang et al., 2020). Jiang’s group discovered that piperine (36A) had anti-HBV activity and could inhibit secretion of hepatitis B virus surface antigen (HBsAg) and hepatitis B virus e antigen (HBeAg), thereby suggesting a *rationale* for development of new drugs that can prevent and treat HBV infection (Jiang et al., 2013).

### 2.6 Structure–activity relationship of alkaloids with respect to virus activity

**FIGURE 2:**
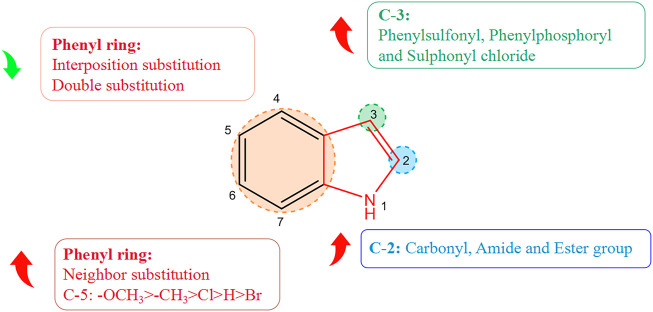
Structure–activity relationship of alkaloids with respect to viruses.

Derivatization of alkaloids with respect to antiviral features had focused mainly on indole alkaloids. Nitrogen-containing heterocycles have shown high antiviral activity. The structure–activity relationship with regard to the antiviral activity of indole alkaloids is summarized in Figure 2, where positions 2, 3, and five of the indole ring are the essential active sites for indole alkaloids to exert antiviral effects. Introduction of hydrophilic groups such as amide, carbonyl, and ester at the 2-position, the phenyl ring at the 3-position terminus, and a halogen group at the 5-position can enhance the antiviral activity of indole alkaloids. Derivative (37A) of indole alkaloids synthesized by Regina and colleagues showed potent activity against HIV-1 reverse transcriptase (RT) and HIV-1 with an IC_50_ value of 1.3 nM (La Regina et al., 2011). Dousson and coworkers revealed that aryl phosphorindole (38A) was a potent non-nucleoside reverse transcriptase inhibitor (NNRTI) of the HIV with an IC_50_ value of 0.34 μM, and that (37A) and (38A) shared a similar pharmacophore profile (Dousson et al., 2016). Hassam and collaborators used a cyclopropylindole derivative as the basic backbone to synthesize NNRTIs of the HIV by introducing amide, carboxyl, and ester groups at the 2-position. Experimental results indicated that the amide and ester groups could enhance the antiviral activity of these compounds. Compound (39A) showed the most potent antiviral activity (IC_50_ = 0.066 µM), whereas the carboxyl group was not as effective in inhibiting the HIV, presumably because of the poor permeability of the carboxyl group, which was ionized at physiological pH (Hassam et al., 2012). Chander and his colleagues derivatized 3-hydroxy-3-(2-oxo-2-phenylethyl)indolin-2-one as a basic backbone and evaluated its anti-HIV-1 activity *in vitro*. Substitution with bromine or chlorine at position 5 (R1) of the oxindole ring enhanced its antiviral activity significantly. Compound (40A) with a chlorine substitution had higher antiviral activity (IC_50_ = 5.92 μM), whereas little antiviral potency was observed in case of substitution of bromine on the oxindole ring with hydrogen (Chander et al., 2018). Moreover, the antiviral activity varied depending on the type and position of the substituents on the benzene ring. The electron-donating methyl (41A), methoxy (42A), and halogenated chlorine groups (43A) increased their antiviral inhibition (IC_50_ = 1.38, 0.82, and 2.03 μM, respectively), with the methoxy group having the most significant antiviral activity. Comparison of the antiviral activity of *o*-substituted (44A) (IC_50_ = 0.76 μM), inter-substituted (45A) (IC_50_ = 34.25 μM), and double-substituted (46A) (IC_50_ = 68.86 μM) revealed that *o*-substitution could strengthen the inhibitory ability of the compounds against viruses, whereas inter-substitution and double substitution had a negative effect on antiviral activity.

## 3 Quinones

### 3.1 Antiviral activity of quinones

**FIGURE 3:**
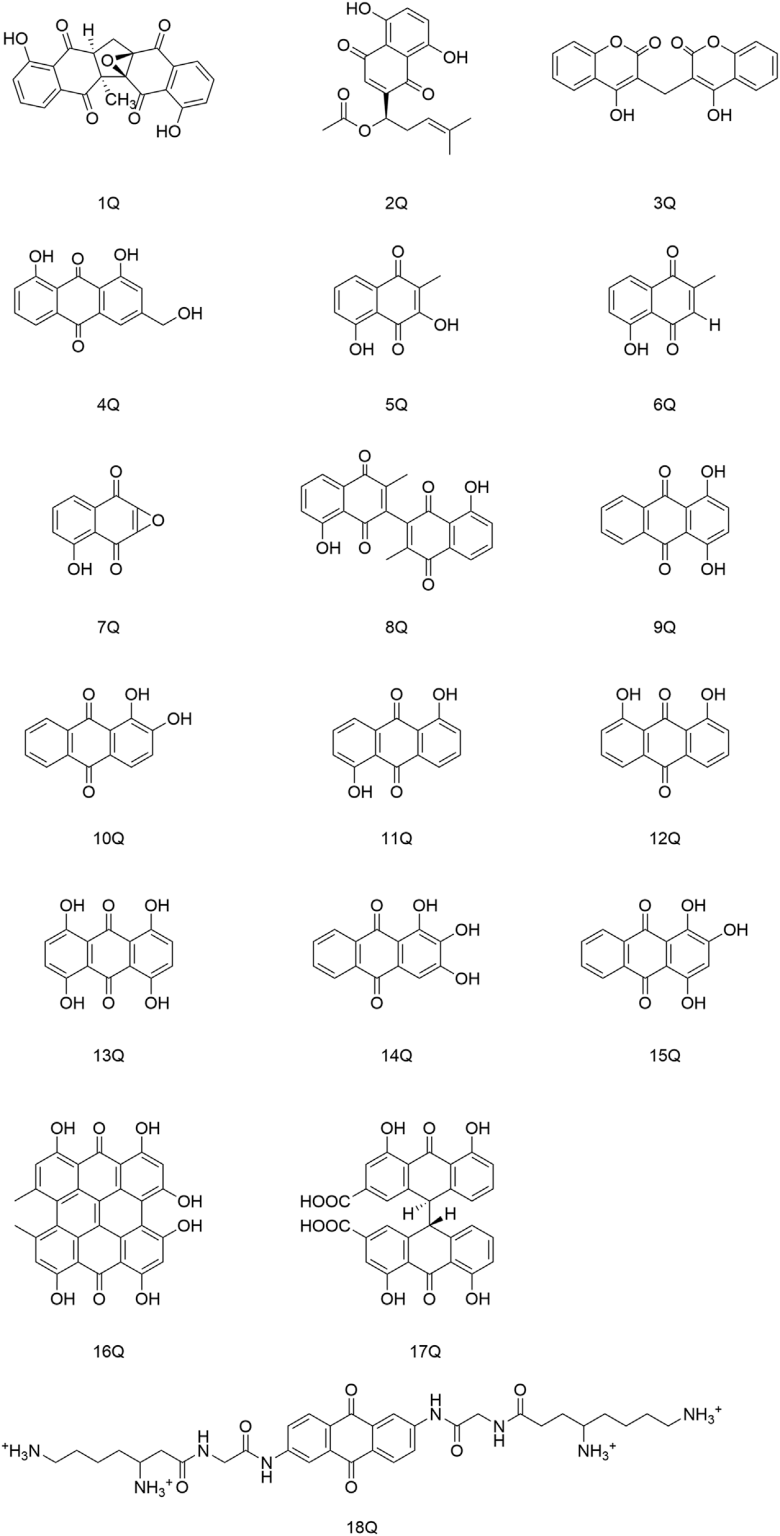
Quinones (and their derivatives) with antiviral activity.

Quinones are a class of aromatic organic compounds with two double bonds and a cyclic diketone structure with six carbon atoms. Quinones can be categorized into four groups based on their structure: benzoquinone, naphthoquinone, anthraquinone, and phenanthrenequinone (Patel et al., 2021), in which the main ones with antiviral activity are anthraquinone and naphthoquinone (Figure 3).

Cetina and coworkers discovered that the naphthoquinone compound zeylanone epoxide (1Q), isolated from *Diospyros anisandra*, could exert activity against influenza-A and -B viruses. Compound (1Q) could reduce viral titers and block the extra-nuclear transport of viral nucleoprotein, and could be a promising drug against influenza viruses (Cetina-Montejo et al., 2019). Liu and his team established that acetylshikonin (2Q) could inactivate viral particles directly at relatively low concentrations to block the uptake or entry of coxsackievirus A16 (CVA16) *in vitro*. Hence, (2Q) could protect cells from CVA16, and inhibit *in vivo* and *ex vivo* infection by CVA16 (Liu X. et al., 2019). Cheng and collaborators identified that dicoumarol (3Q) could inhibit the transcription of covalently closed circular-DNA by promoting degradation of the targeted viral protein (HBx), thereby combating chronic infection with the hepatitis B virus (Cheng S. T. et al., 2021). Parvez and his colleagues identified the potential of aloe-emodin (4Q) in hepatocellular carcinoma cells, likely through inhibition of the polymerase activity of the HBV (Parvez et al., 2019).

### 3.2 Structure–activity relationships of quinones with respect to viruses

**FIGURE 4:**
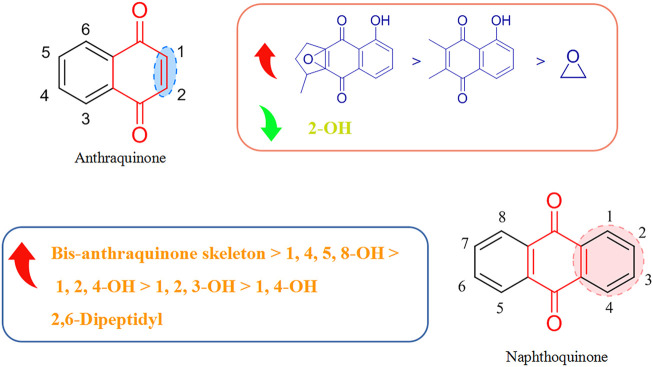
Structure–activity relationship of quinones with respect to viruses.

Most of the quinones that display antiviral activity are naphthoquinone and anthraquinone compounds, and the structure–activity relationship of their antiviral effects is depicted in Figure 4.

Montejo’s team observed that the naphthoquinone compound droserone (5Q) possessed weak activity against influenza viruses as well as cytotoxicity. Plumbagin (6Q) (in which the 2-position hydroxyl group is replaced with hydrogen) showed antiviral activity, and it was assumed that the 2-position hydroxyl group inhibited the antiviral activity of naphthoquinone (Cetina-Montejo et al., 2019). However, 2,3-epoxiplumbagin (7Q) and the naphthoquinone dimer 3,3-biplumbagin (8Q), which are structurally similar and contain an epoxide structure, reduced their cytotoxicity to different degrees, and (1Q) (which has an epoxide structure and a naphthoquinone backbone) showed the most significant activity against influenza viruses, with an IC_50_ value of 0.65 ± 0.01 µM. They hypothesized that the presence of epoxide structures and naphthoquinone multimers in naphthoquinone compounds could enhance their antiviral activity.

Thus, the antiviral activity of anthraquinones appears to be related to the number and location of phenolic hydroxyl groups in their structures. Also, formation of a keto-phenol system on the same benzene ring is the key to their antiviral activity. Furuta and his colleagues showed that derivative (9Q) inhibited hepatitis C virus (HCV) replication (IC_50_ = 54 µM) mainly by suppressing the activity of NS3 decarboxylase. The activity of (9Q) was superior to that of (10Q), (11Q), or (12Q) (Furuta et al., 2015). Also, increasing the number of hydroxyl groups on the same benzene ring and the number of pairs of keto-phenol systems could further improve the inhibitory activity. They found that (13Q) had the most potent inhibitory activity (IC_50_ = 6 µM), and that (14Q) and (15Q) had similar abilities to inhibit NS3 decyclase, with IC_50_ values of 18 and 11 μM, respectively. Anti-HCV activity was also augmented significantly by multimerization of hydroxyanthraquinones, such as (16Q) and (17Q), both of which had a double-anthraquinone backbone structure with IC_50_ values of three and 0.8 µM, respectively. In addition, the antiviral activity of anthraquinones might be potentiated to some extent by insertion of a group capable of inhibiting the activity of viral proteins into the anthraquinone structure. Frecentese and coworkers discovered that positions two and six of the anthraquinone ring were crucial for the synthesis of HIV-1 nucleocapsid inhibitors, and synthesized the compound (18Q), which provided the groundwork for development of new anti-HIV drugs (Frecentese et al., 2016).

## 4 Flavonoids

“Flavonoids” is a general term for compounds with a C6-C3-C6 skeleton based on 2-phenylchromanone as the parent nucleus (Liu et al., 2021). Flavonoids can be divided into flavonoids, flavonols, isoflavones, and dihydroflavonoids according to the degree of oxidation of the C3 chain and position of the benzene-ring linkage (Fang et al., 2015).

**FIGURE 5:**
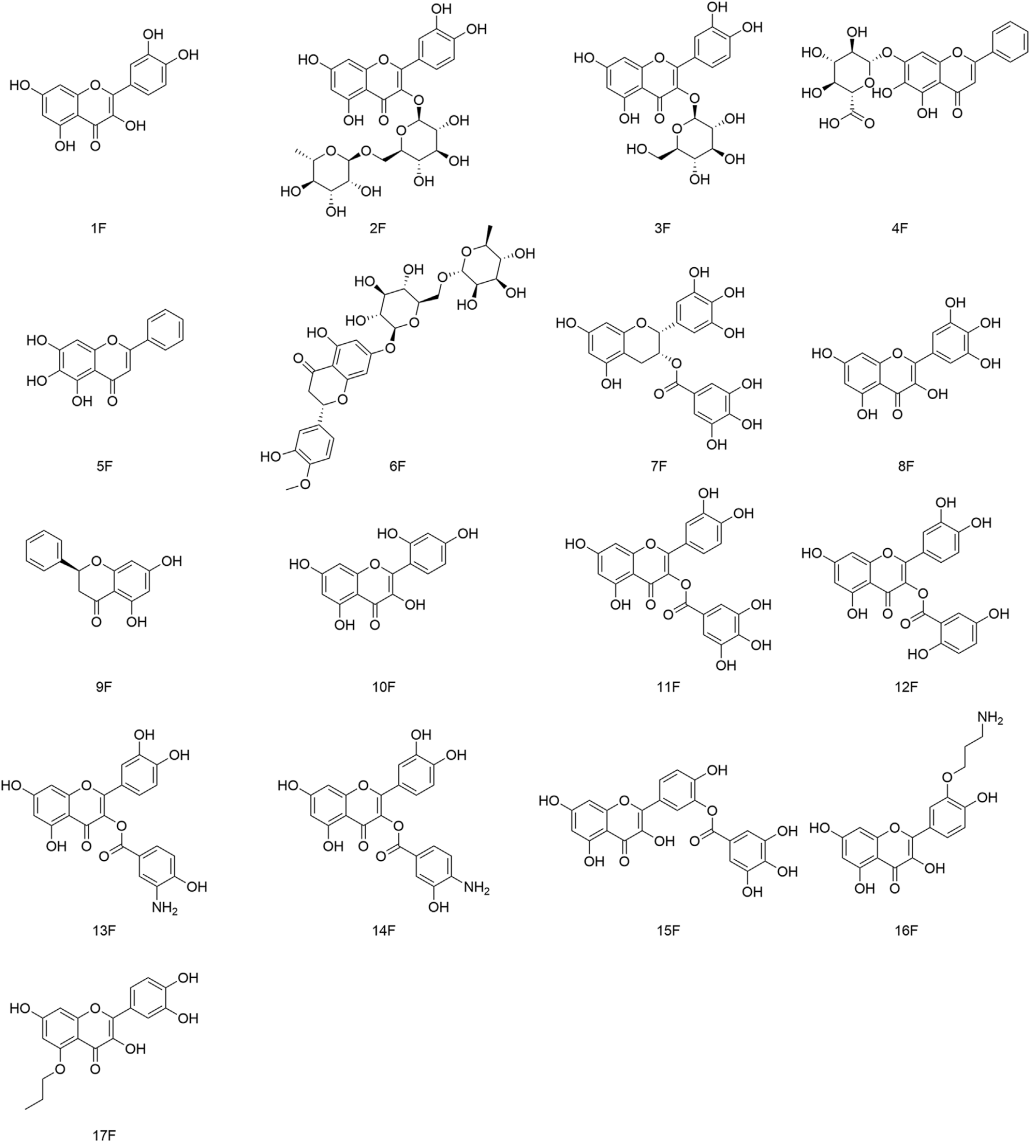
Flavonoids (and their derivatives) that possess antiviral activity.

Zandi and his team showed that flavonoids have activity against DENV-2 in Vero cells. Autophagy, the inflammation-related nuclear factor-kappa B pathway, and Toll-like receptor pathway might be the major molecular targets of flavonoids against viruses (Zandi et al., 2011; Cheng C. et al., 2021). We have described some representative flavonoids with significant antiviral activity in this review. The structures of flavonoids (and their derivatives) that possess antiviral activity are shown in Figure 5.

### 4.1 Flavonoids and flavonols

#### 4.1.1 Quercetin and rutin

Quercetin (1F) is the most common flavonol compound with notable antiviral effects found in nature (Li et al., 2016). Xu and coworkers demonstrated that (1F) had good protective effects against the cardiomyocyte damage wrought by CVB3 infection. Shohan and collaborators used (1F) in combination with the antiviral drugs raltegravir and famipiravir to treat critically ill inpatients with neocoronary pneumonia, and (1F) showed a more significant effect than that observed using raltegravir alone or famipiravir alone (Xu et al., 2021; Shohan et al., 2022). Rutin (2F) is a flavonol ligand composed of (1F) and rhamnoglucoside. (2F) has been shown to exert activity against the HBV, influenza viruses, human noroviruses, and the DENV (Li K. et al., 2021). Kim and her colleagues investigated the antiviral activity of (1F), (2F), and isoquercetin (3F) against influenza-A and B viruses. (3F) showed the highest antiviral activity (ED_50_ = 1.2 µM), even better than that of the positive control drug amantadine (ED_50_ = 1.4 µM) (Kim et al., 2010).

#### 4.1.2 Baicalin and baicalein

Baicalin (4F) is a glycoside flavonoid with high polarity. Baicalein (5F) is the aglycone part of (4F). Lani’s team revealed that (5F) had stronger intracellular antiviral activity in the post-entry phase of CHIKV replication, with an IC_50_ value of 1.891 μg/ml and selectivity index (SI) of 188.4, much stronger than that of the positive control, ribavirin (IC_50_ = 11.07 μg/ml, associated SI = 54.2) (Lani et al., 2016).

(5F) also possesses anti-CHIKV activity, in which the hydroxyl group at position seven on the baicalin ring A is replaced with a glucouronoid (EC_50_ = 7 μM). It inhibits different stages of the replication cycle of the CHIKV as well as the production and expression of CHIKV protein, thereby eliciting direct viral killing (Oo et al., 2018). Zhu’s group showed that (5F) had anti-influenza virus A3/Beijing/30/95 (H3N2) activity, mainly through inhibition of formation of the autophagy-related gene 5 (Atg5)–autophagy-related gene 12 (Atg12) complex and autophagy-related protein light chain 3 (LC3-II) expression, as well as reducing virus replication by suppressing the influenza virus-induced autophagy pathway (Zhu et al., 2015).

### 4.2 Other flavonoids

Hesperidin (6F) is a glycoside formed by hesperetin and rhamnoglucoside. (6F) is a dihydroflavonoid derivative. Meneguzzo and colleague suggested that (6F) could interfere with different stages of the invasion and replication of coronaviruses. (6F) has extremely strong binding capacity to the receptors for SARS-CoV-2 (Meneguzzo et al., 2020). Epigallocatechin-3-gallate (EGCG) (7F) is a major component of tea. Pang and colleagues observed that (7F) had anti-HBV activity. Treatment of HepG2 2.2.15 cells with (7F) (50 μg/ml) for 6 days could repress secretion of HBsAg and HBeAg significantly (53% and 44% inhibition, respectively) and inhibition of HBsAg was stronger than that of the positive control lamivudine (Pang et al., 2014).

### 4.3 Structure–activity relationship of flavonoids with respect to viruses

**FIGURE 6:**
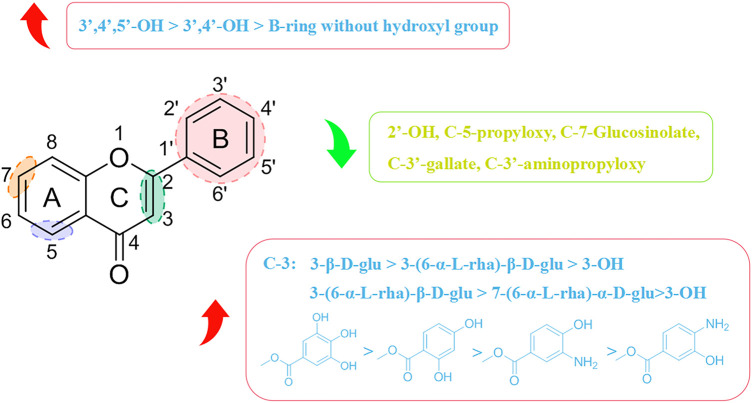
Structure–activity relationships with respect to the antiviral activity of flavonoids.

Most flavonoids possess a C6-C3-C6 skeleton. The type and position of substituent groups can affect their antiviral activity. The specific structure–activity relationships are shown in Figure 6. Pasetto and his team discovered that myricetin (8F) had the highest activity against HIV-1 *in vitro* (IC_50_ = 20.43 µM), which was about four-times that of (1F) (IC_50_ = 88.98 µM) and 16-times that of pinocembrin (9F) (IC_50_ = 346.75 µM) under identical conditions (Pasetto et al., 2014). (8F) has 3′, 4′, and 5′ hydroxyl groups, whereas (1F) has two adjacent hydroxyl groups at 3′ and 4′ positions, and no hydroxyl group is present in any of these positions in (9F). The relationship between their structure and antiviral activity has been hypothesized to be 3′,4′,5′-OH > 3′,4′- OH > B-ring without OH. The greater the number of hydroxyl groups on the B-ring, the more potent is the antiviral activity of flavonoid compounds. Besides the number of hydroxyl groups on the B-ring, the position of hydroxyl groups on the B-ring can also influence their antiviral activity. Morin (10F) and (1F) are flavonol compounds containing two free hydroxyl groups on the B-ring, but they are present in different positions, with (10F) having a 2′,4′ interposition dihydroxy group and (1F) having a 3′,4′ neighboring dihydroxy group. Carvalho’s group revealed that the anti-Canine distemper virus (CDV) activity of mulberry pigment was weaker than that of (1F). They speculated that the 2′ hydroxyl group on the B ring might influence its antiviral activity (Carvalho et al., 2013). Tahpa’s group modified the C-3, C-5, and C-3′ hydroxyl groups on (1F). They concluded that introduction of gallate, dihydroxybenzoate, and aminohydroxybenzoate at C-3 improved the antiviral activity of (1F), with (11F) showing the most potent antiviral activity (ED_50_ = 9.1 µM), which was similar to (4F) activity (ED_50_ = 8.3 µM). In contrast, introduction of gallate, aminopropoxy, and propoxy at C-5 and C-3′ curtailed the antiviral activity of (1F), presumably because 3′-OH and 5-OH were the active groups involved in the antiviral action of (1F) (Thapa et al., 2012).

Flavonoids are combined with carbohydrates to form glycosides in plants. The linkage position and type of sugar affects their antiviral activity. Carvalho and his colleagues showed that both (2F) and (7F) had stronger anti-CDV viral activity than (1F) (Carvalho et al., 2013). They postulated that the glycosylation of (2F) and (7F) could enhance their antiviral activity, and that the degree of improvement in antiviral activity was related to the glycosylation site, with (2F) glycosylation at C-3 being distinctly superior to (7F) glycosylation at C-7. Thapa and collaborators demonstrated that (3F) containing 3-β-D-glu had considerable antiviral activity (ED_50_ = 1.2 µM), which was superior to that of (2F) containing 3-(6-α-L-rha)-β-D-glu (ED_50_ > 100 µM) (Thapa et al., 2012). However, not all flavonoid glycosides have stronger antiviral activity than their aglycones. In terms of activity against DENV-2, (5F) is weaker than (6F). 7-OH might be an important moiety for the antiviral activity of (6F) (Moghaddam et al., 2014).

## 5 Terpenoids

**FIGURE 7:**
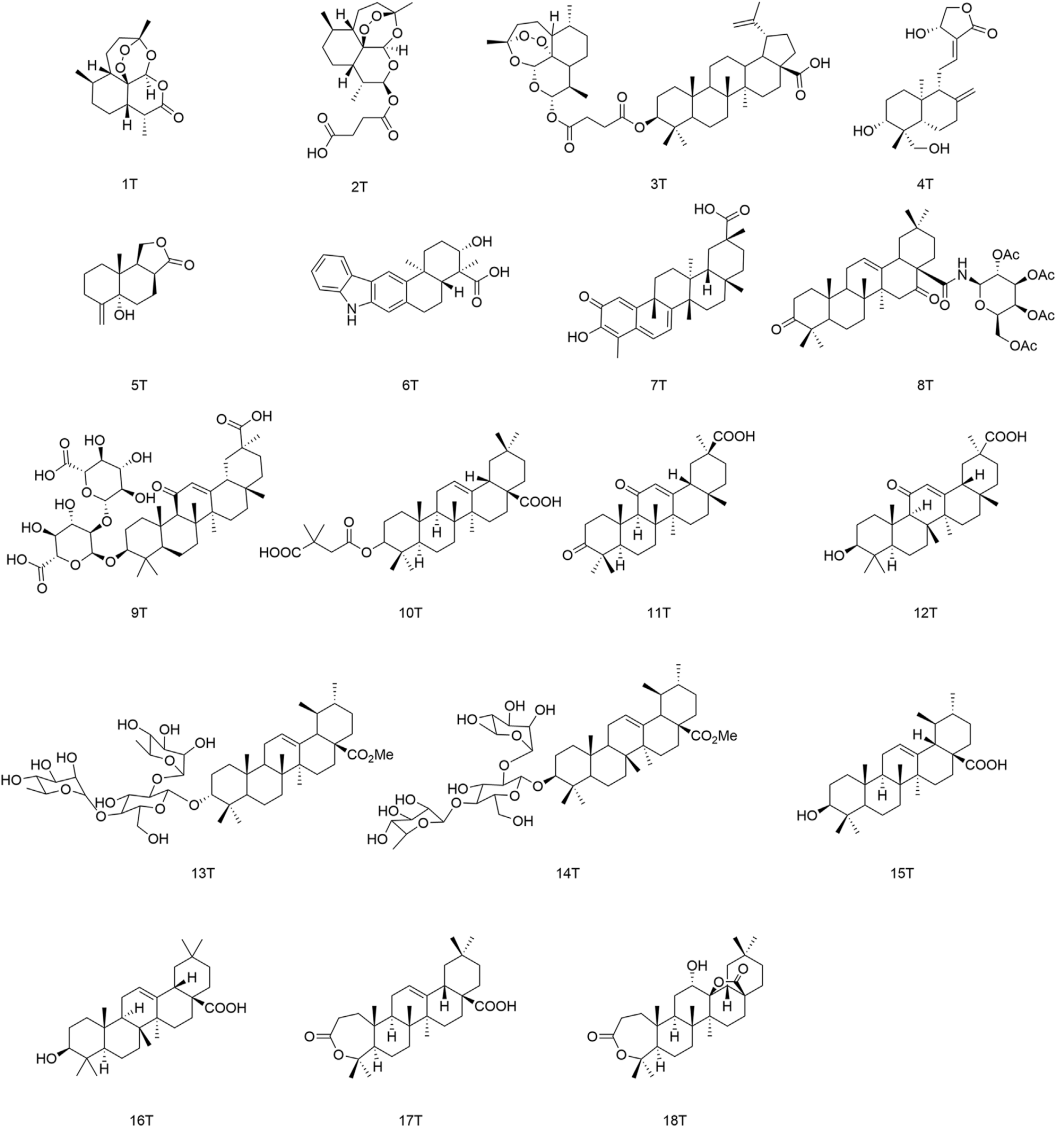
Terpenoids (and their derivatives) with antiviral activity.

Terpenoids are a group of hydrocarbons occurring naturally in plants. They can be classified as monoterpenes, sesquiterpenes, triterpenes, and polyterpenes according to the number of isoprene units in the molecule (Zhang et al., 2018). Thanks to research into new antiviral drugs, the essential oils of plants have become popular due to their high efficiency, safety, and resistance (Zhang et al., 2021). The structures of terpenoids with antiviral activity are presented in Figure 7.

### 5.1 Monoterpenes and sesquiterpenes

The monoterpenes present in the essential oils of plants include monoterpene alcohols and monoterpene aldehydes, which having slightly higher antiviral activity than monoterpene alcohols (Astani et al., 2010). Artemisinin (1T) is a sesquiterpene lactone, and its derivatives have shown inhibitory effects against the human cytomegalovirus (HCMV), HBV, and HCV. In particular, artesunate (2T) can control secretion of HBsAg with an IC_50_ of 2.3 μmol/L and reduce gene expression of the HBV with an IC_50_ of 0.5 μmol/L (Wohlfarth and Efferth, 2009). Karagoz and collaborators showed that derivative (3T) presented high anti-HCMV activity (EC_50_ = 0.24 µM), which was 15-times higher than the antiviral activity of betulinic acid and 23-times that of (2T), as well as being superior to the clinically used anti-HCMV drug ganciclovir (Karagoz et al., 2019). Panraksa and colleagues showed that andrographolide (4T) displayed appreciable anti-DENV activity in Hep G2 and HeLa cells, with EC_50_ values of 21.304 and 22.739 µM, respectively (Panraksa et al., 2017). Liu and his team identified a new 14-demethylamino-based sesquiterpene, phomanolide (5T), with high activity against influenza A virus (HIN1) (IC_50_ = 2.96 ± 0.64 μg/ml), which was first isolated from *Aconitum vilmorinianum.* (Liu S. S. et al., 2019). Ding et al. isolated a pentacyclic indole sesquiterpene named xiamycin (6T) from *Streptomyces* species with moderate anti-HIV activity. (6T) blocked the entry of C-C chemokine receptor 5 (CCR5)-tropic HIV-1, indicating that the pentacyclic carbazole system might be an effective backbone for antiviral agents (Ding et al., 2010).

### 5.2 Triterpenes

Triterpenoids are composed mainly of six isoprene units, of which pentacyclic triterpenes are the most common and exhibit strong antiviral activity. The main types of pentacyclic triterpene skeletons are oleanolane, ursolidane, lupinane, and corkolidane (Miranda et al., 2022).

Tseng and coworkers showed that celastrol (7T) could induce gene expression of heme oxygenase-1, which led eventually to HCV inhibition (Tseng et al., 2017). Si and collaborators discovered that (8T) (a derivative of echinocystic acid combined with acetylated galactose) exerted prominent effects against the Ebola virus, with IC_50_ values of 59.2 ± 1.6 nM (Si et al., 2018). Matsumoto and colleague demonstrated that glycyrrhizin (9T) possessed anti-HCV activity (EC_50_ = 16.5 µM) and that its mechanism of action involved controlling the release of infectious HCV particles (Matsumoto et al., 2013).

### 5.3 Structure–activity relationships of pentacyclic triterpenoids with respect to viruses

**FIGURE 8:**
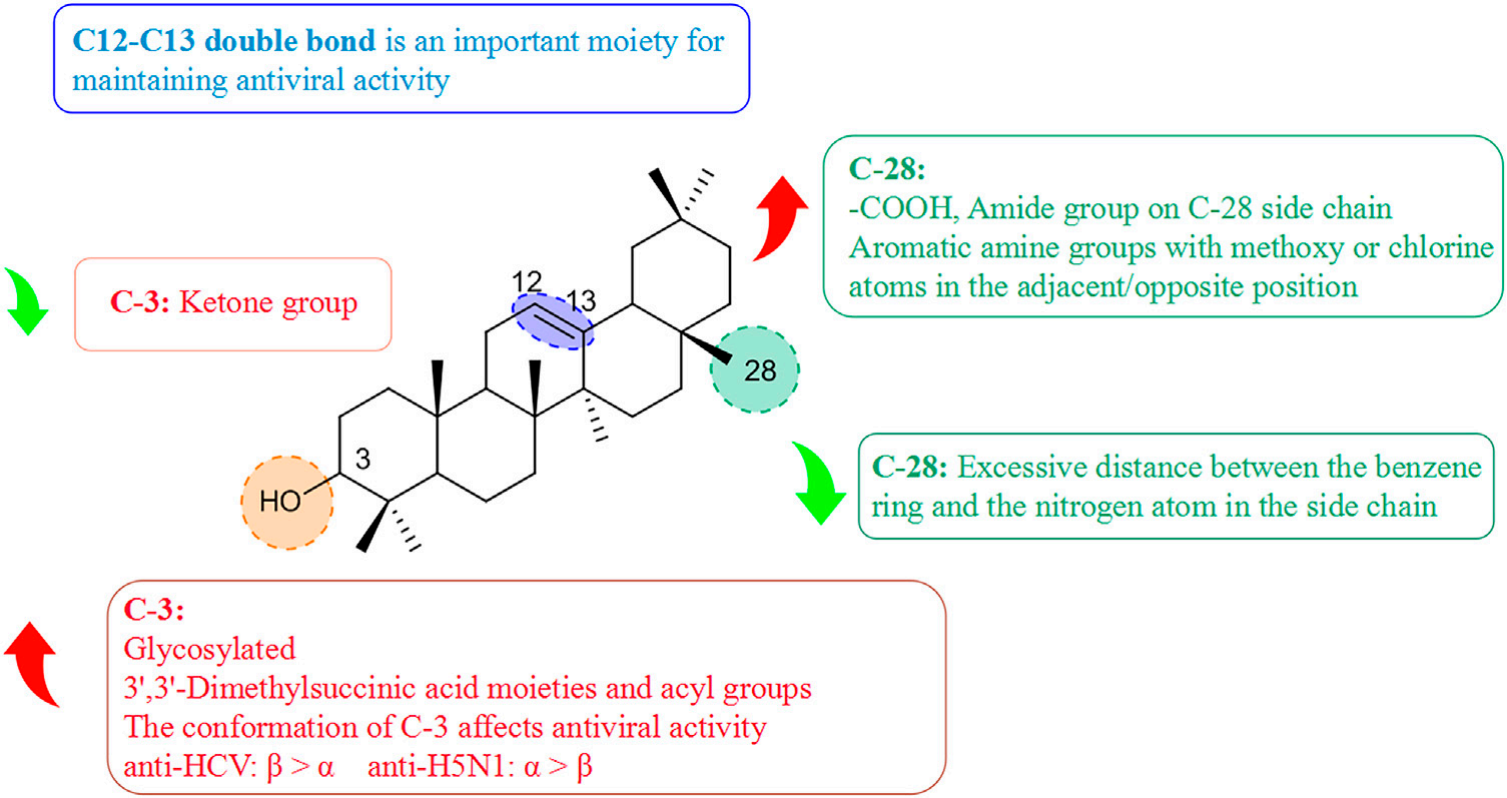
Structure–activity relationship of pentacyclic triterpenoids with regard to viruses.

Pentacyclic triterpenoids influence antiviral activity mainly at C-3, C12–C13, and C-28 positions (Fan et al., 2020) (Figure 8). Introducing of glycosyl groups, 3′,3′-dimethylsuccinic acid, and acyl groups at C-3 can enhance antiviral activity. Cai’s group observed that the pentacyclic triterpene parent nucleus and glucose molecules were essential in upgrading the activity of compounds against influenza viruses (Cai et al., 2022). Yu and collaborators concluded that derivative (10T) containing a 3′,3′-dimethylsuccinic acid moiety had an EC_50_ value of 0.32 µM against HIV-1, whereas derivatives containing 3′,3′-dimethylpentanedioic acid showed no antiviral activity (Yu et al., 2006). Wang and colleagues reported that (11T) was oxidized to a ketone group, and its ability to inhibit secretion of HBsAg protein (IC_50_ = 432.54 µM) proved to be much weaker than that of glycyrrhetinic acid (12T) (IC_50_ = 20.86 µM), presumably because the 3-ketone group suppresses the antiviral activity of triterpenoids (Wang L. J. et al., 2012). Moreover, the C-3 configuration proffered different advantages in the prevention of different viral species. Ma and his team demonstrated that β-configuration substituent-containing derivatives of oleanolic acid were superior to the α-configuration counterparts in terms of anti-HCV activity (Ma et al., 2009). Song’s team revealed that (13T) containing an α-configuration hydroxyl group could maintain activity against influenza A virus (H5N1) and reduce cytotoxicity against MDCK cells greatly, stronger than the β-configuration (14T) (Song et al., 2015).

The free hydroxyl group at the C-3 position and free carboxyl group at the end of the side-chain at the C-28 position are crucial moieties for the antiviral activity of triterpenoids (Sun et al., 2002) such as ursolic acid (15T) and oleanolic acid (16T), which both showed high anti-HCV activity (Kong et al., 2013). Liao and colleagues discovered that introducing of an amide group in the side-chain at the C-28 position was beneficial for enhancing antiviral activity and reducing cytotoxicity. The antiviral activity of aromatic amine derivatives was obviously better than that of aromatic methylamines, which suggests that the distance between the benzene ring and nitrogen atom is too long to depress antiviral activity (Liao et al., 2019). It was possible to improve the anti-H5N1 activity of aromatic amine compounds containing methoxy or chlorine atoms substituted at adjacent/opposite positions in the side-chain in preference to those containing inter-substituted aromatic amines. Li’s team synthesized a series of triterpenoid derivatives of 3,4-lactones, among which derivative (17T) with a C12–C13 double bond developed stronger inhibition of secretion of the HBV protein HBeAg (IC_50_ = 0.86 µM), whereas the antiviral activity of (18T) with an oxidized double bond decreased (IC_50_ = 149.1 µM), thereby suggesting that the C12–C13 double bond played an important role in the maintenance of activity (Li et al., 2018).

## 6 Polysaccharides

Polysaccharides are natural macromolecules with a wide range of origin. In general, polysaccharides consist of >10 monosaccharide molecules that have been polymerized, which contain multiple chiral centers and most are non-cytotoxic (Muralidharan et al., 2019). Polysaccharides and their derivatives display prominent suppressive effects against the HIV, HSV, enteroviruses, and influenza viruses, and become a focus of research (Wang C. R. et al., 2011; Saha et al., 2012; de Godoi et al., 2014; Wu et al., 2016).

### 6.1 Plant-derived polysaccharides

Plants are the main natural source of polysaccharides. Oliveira and coworkers found that the crude aqueous and alkaline extracts of *Stevia rebaudiana* leaves possessed activity against HSV-1 *in vitro* (de Oliveira et al., 2013). Ceole and collaborators noticed that anti-HSV-1 activity was more pronounced in the crude fraction, which was related directly to the interaction between the *S. rebaudiana*-derived polysaccharide and viral glycoprotein, not to cellular receptors (Ceole et al., 2020). Su’s team demonstrated that distilled-water and 95%-ethanol extracts of *Ardisia chinensis* Benth exerted varying degrees of activity against CVB3 *in vitro*, with the aqueous extract being more active (IC_50_ = 3.9 μg/ml) (Su et al., 2006). This antiviral activity was derived mainly from a neutral polysaccharide with d-glucose as the main glycoside.

#### 6.1.1 Ginseng polysaccharides

Baek and colleagues showed that two ginseng pectin polysaccharides suppressed rotavirus-induced cell death in a dose-dependent manner. They inhibited the binding of rotaviruses to host cells (IC_50_ = 15 and 10 μg/ml), with the hairy region possibly being its functional site (Baek et al., 2010). Yoo’s group showed that ginseng polysaccharides boosted the survival of H1N1- and H3N2 influenza-infected mice, demonstrating that ginseng polysaccharides could be used as therapeutic agents against infections by influenza viruses (Yoo et al., 2012).

#### 6.1.2 *Houttuynia cordata* polysaccharides

Cheng and his team revealed that *H. cordata* polysaccharides possessed activity against human noroviruses by deforming and swelling viral particles, thereby inhibiting virus penetration into target cells (Cheng et al., 2019). Zhu and coworkers found that treatment with *H. cordata* polysaccharides could improve the survival chances of mice infected with IAV-H1N1, protecting them from lung and intestinal damage as well as reducing viral replication. *H. cordata* polysaccharides might have potential as an alternative drug for treatment of human IAV infection (Zhu et al., 2018).

#### 6.1.3 Other polysaccharides

Kim’s team discovered that one polysaccharide from dried roots of *Sanguisorba officinalis* was efficient in treatment of Enterovirus 71 (EV71) infections (Kim et al., 2022). Vinicius and coworkers found that polysaccharides from *Leptospermum* species could influence the initial replication of poliovirus type 1 and bovine herpes zoster virus and had high antiviral activity (Rincao et al., 2012). Lin and collaborators identified a polysaccharide fraction in *Platycladus orientalis (L.) Franco* with anti-HBV activity, primarily via repression of expression of HBsAg and HBeAg and interfering with replication of HBV DNA, with IC_50_ values of 1.33 ± 0.12, 1.67 ± 0.13, and 0.80 ± 0.03 mg/ml, respectively (Lin et al., 2016).

### 6.2 Sulfated derivatives

Sulfated polysaccharides are natural and semi-synthetic acidic polysaccharides formed by substitution of a hydroxyl group for a sulfate group on a monosaccharide in a macromolecular chain (Lu et al., 2021). Usually, sulfated polysaccharides have high activity because the negatively charged sulfate group can bind to glycoproteins in the viral envelope, thereby prohibiting the viral particle from binding to and penetrating the target cell. Sulfated modifications appear to be critical for polysaccharides, with sulfated polysaccharides having greater potential for antiviral activity. For example, the sulfated polysaccharides from *Auricularia auricula* and *Tremella* species have strong activity against the Newcastle disease virus (Zhao et al., 2011; Nguyen et al., 2012). Ma and coworkers isolated a new partially sulfated polysaccharide, PSP-2B, with low cytotoxicity and activity against HSV-1 (IC_50_ = 69 μg/ml) and HSV-2 (IC_50_ = 49 μg/ml) (Ma et al., 2016). Galhardi and colleagues evaluated the activity of *Azadirachta indica* polysaccharides (P1 and P2) and their sulfated derivatives (P1S and P2S) against the poliovirus and herpes zoster virus: P1S was the most active and interacted mainly in the initial stages of viral replication (Faccin-Galhardi et al., 2012). Godoi and collaborators investigated the activity of sulfated polysaccharides from *Adenanthera pavonina* seeds against poliovirus type 1, and concluded that they repressed poliovirus type 1 at several steps of replication and had low cytotoxicity (de Godoi et al., 2014). LJ04 is an acidic polysaccharide that can inactivate EV71 within 2 h at 37°C (Yue et al., 2017). The sulfate group is vital to the antiviral activity of LJ04 (Li et al., 2020). Mukherjee and collaborators chemically vulcanized arabinoxylan (1P) from the seed husks of *Plantago ovata* and found that the sulfate group of arabinoxylan (2P) conferred activity against HSV-1 (Mukherjee et al., 2021). Kappa carrageenan (3P) is present in red algae plants. Kappa carrageenan (3P) and its sulfated derivatives have high inhibitory effects against IAV replication *in vitro* and *in vivo* (Wang W. et al., 2012). Oral or nasal sprays containing kappa gum have been shown inactivate SARS-CoV-2 infection in cultures of human airway epithelial cells (Schutz et al., 2021).

### 6.3 Structure–activity relationship of polysaccharides with regard to viruses

The type of functional group in polysaccharides is closely related to their antiviral activity. Cai’s team demonstrated that pectic polysaccharides derived from the above-ground parts of *Portulaca oleracea L.* could restrain viral penetration and possessed anti-HSV-2 activity. It has highly methyl-esterified and partially acetylated residues of galacturonic acid in its structure. Its anti-HSV-2 activity ceases after removal of esterification; the methyl esterification or acetylation of galacturonic acid (GalA) residues might be responsible for the antiviral effect (Dong et al., 2010). Liu and collaborators subjected neutral polysaccharides extracted from *Polygonatum cyrtonema Hua* to sulfation, phosphorylation, carboxymethylation, acetylation, or sulfonylation. Phosphorylation or sulfation could intensify the inhibitory activity of neutral polysaccharides against the HSV. The sulfonylated derivative had identical activity to that of neutral polysaccharides. Acetylation or carboxymethylation depressed the antiviral activity of neutral polysaccharides (Liu et al., 2011).

**FIGURE 9:**
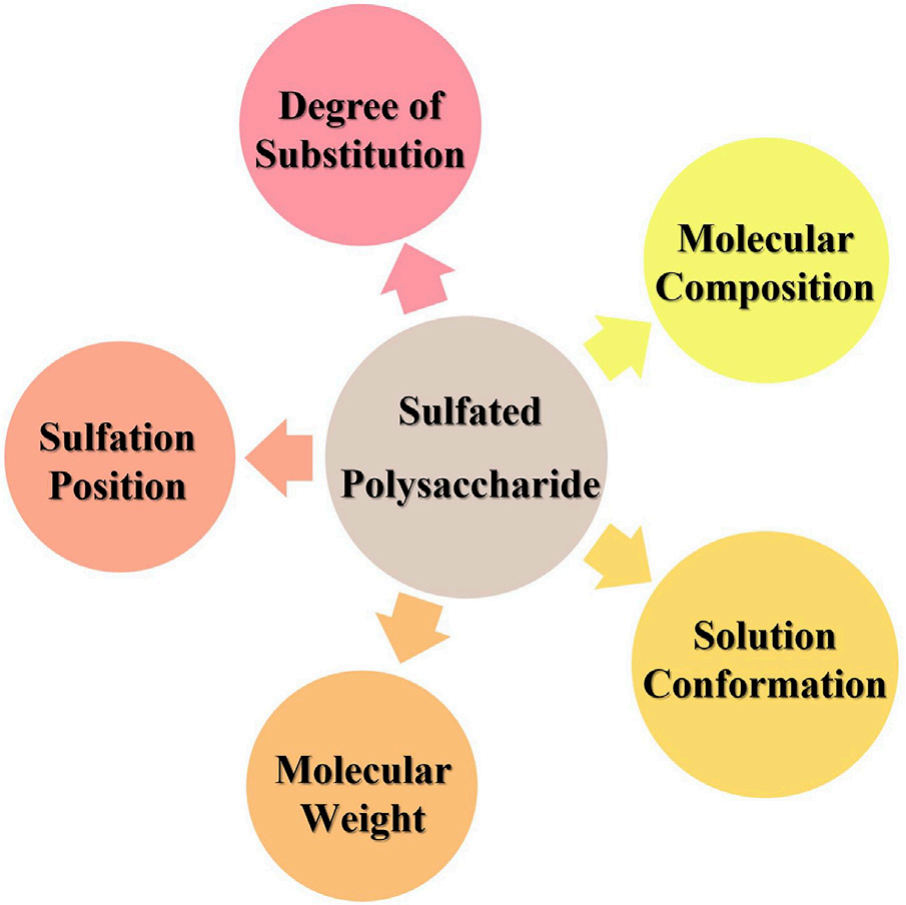
Factors affecting the antiviral activity of sulfated polysaccharides.

Sulfation is the most common approach to chemical derivatization of polysaccharides. Various factors influence the antiviral activity of sulfated polysaccharides: the degree of substitution, sulfation position, molecular composition, molecular weight, and solution conformation of the polysaccharide (Ghosh et al., 2009) (Figure 9). The number of sulfate groups is correlated closely with antiviral activity in polysaccharides. Jiao and his colleagues isolated four polysaccharides from several Atlantic Canadian seaweeds, all of which had activity against influenza viruses. The activity of these four polysaccharides decreased as their sulfate content increased (Jiao et al., 2012). However, the antiviral activity of the polysaccharides did not follow a simple linear relationship with the degree of sulfation. Wang’s group revealed that the anti-IAV activity of carrageenan oligosaccharides was significantly different despite possessing similar sulfate content. K-keratan gum oligosaccharide had the highest activity at a sulfate content of 0.8–1.0 mol/mol of disaccharide and a molecular weight of 1–3 kDa, thereby indicating that sulfation sites also influenced antiviral activity (Wang W. et al., 2012). Thuy and coworkers reported that fucoidan isolated from three species of brown seaweed possessed distinctive anti-HIV activity. However, the anti-HIV activity of compounds with different degrees of sulfation and sulfate sites was very similar to each other, which suggested that the molecular weight and type of glycosidic bond of fucoidan were the main factors affecting their antiviral activity (Thuy et al., 2015).

In addition, the molecular weight and conformational changes of a polysaccharide can affect its antiviral activity. Witvrouw and his team revealed that dextran sulfate of higher molecular weight had higher antiviral activity than that of dextran sulfate of lower molecular weight if the molecular-weight range was 1–500 kDa (Witvrouw and De Clercq, 1997). Lopes and collaborators analyzed the activity of seven chemically modified sulfated polysaccharides from green seaweed against the HSV. SU1F1 (a heterosaccharide with a molecular weight of 34 kDa) provided clearly superior antiviral activity to that of SU1F2 (molecular weight <5 kDa) (Lopes et al., 2017). Tuvaanjav’s group sulfated two water-soluble polysaccharides from *Cynomorium songaricum Rupr.* They noted that the sulfated polysaccharide could inhibit HIV infection with an EC_50_ value of 0.3–0.4 μg/ml. They postulated that sulfation changed the conformation of the polysaccharide and enhanced the electrostatic interaction of sulfate groups (Tuvaanjav et al., 2016).

## 7 Organic acids

The main organic acids involved in the antiviral activity of NPs are ferulic acid, cinnamic acid, chlorogenic acid, and caffeic acid.

Carvalho and his colleagues discovered that cis-cinnamic acid (1R) and ferulic acid (2R) had high anti-CDV activity, and that the antiviral effect of ferulic acid (2R) was stronger than that of (1R) (Carvalho et al., 2013). *Trans*-cinnamic acid (3R) is structurally similar to (2R), but did not show activity against the CDV, probably owing to the substituents at the C-4 and C-5 positions of (2R). Chlorogenic acid (4R) had antiviral activity, especially in fighting the herpes virus and CVB3 (Yu, 2017). Ding’s group discovered that (4R) exhibited activity against influenza viruses, with EC_50_ values of 44.87 μM against the H1N1 virus and 62.33 μM against the H3N2 virus (Ding et al., 2017). Caffeic acid (5R) is a degradation product of (4R) and appears to be widespread in plants (Boerjan et al., 2003). Shen and his collaborators reported that (5R) could inhibit HCV replication by activating the Kelch-like ECH-associated protein 1/Nuclear factor (erythroid-derived 2)-like 2 (Keap1/Nrf2) pathway and led to an increase in protein expression of p62, with an IC_50_ value of 100 ± 20 μM (Shen et al., 2018). Ogawa and coworkers found that (5R) could inhibit severe fever with thrombocytopenia syndrome virus (SFTSV) infection with an IC_50_ of 0.048 mM. The mechanism of action was mainly through suppression of binding of the SFTSV to cells rather than dependence upon its acidity (Ogawa et al., 2018). Weng’s team discovered that the activity of (5R) was higher than (4R) in elderberry extracts against human coronavirus NL63, with an IC50 of 3.54, and 43.45 µM, respectively (Weng et al., 2019).

## 8 Others

Tatanan A (1O) is a novel sesquiterpene lignan. It was first identified in the ethanol extract of *Acorus calamus L.* roots (Ni et al., 2011). Yao and colleagues found that (1O) could oppress the translation and early RNA synthesis of DENV-2, thereby resulting in new activity against DENV-2 (EC_50_ = 3.9 µM) (Yao et al., 2018). Cui and collaborators demonstrated that manassantin B (2O) (a lignan-like compound derived from the roots of *Saururus chinensis*) conferred high activity against replication of the Epstein-Barr virus (EBV) with an EC_50_ of 1.72 µM, thereby providing the first evidence of an anti-EBV effect in lignans (Cui et al., 2014). Pang and coworkers reported the anti-HBV activity of lutein (3O). It blocked secretion of HBsAg and the amount of extracellular HBV DNA in HepG2 cells in a dose-dependent manner (Pang et al., 2010). Ratnoglik and coworkers characterized the high anti-HCV activity of pyropheophorbide (4O) from *Morinda citrifolia* leaves. It induced inhibition of RNA replication and protein synthesis of the HCV with antiviral effects at entry and post-entry steps with an IC_50_ of 0.2 μg/ml (Ratnoglik et al., 2014). An enhanced interferon-α (IFN-α) anti-HCV agent, diosgenin (5O) (steroidal saponin of plant origin) was identified by Wang and collaborators as having anti-HCV activity with an EC_50_ of 3.8 μM. A possible mechanism of action may be related to inhibition of expression of signal transducer and activator of transcription-3 (Wang Y. J. et al., 2011).

## 9 Conclusion

Viruses pose a serious challenge to the health and quality of life of humans. Their general spread and rapid mutation has severely compromised the efficacy of antiviral drugs, thereby stimulating research and development of new antiviral drugs (Owen et al., 2022).

Antiviral drugs were developed to be used as a “second step” after vaccination. Use of antiviral agents enables rapid clinical use during outbreaks of viral diseases if vaccines are not available. This strategy can control the spread of viral diseases and protect the lives and health of humans. Vaccine are designed to be virus-specific and to treat individual viruses, but fail to deliver the full range of antiviral effects, including low (or no) effects against mutant strains of a virus (Jefferson et al., 2014). Drug resistance is also an emerging problem. For instance, almost all prevalent influenza strains are mutated with resistance to adamantanes, which suggests an urgent need to reinvigorate development of antiviral drugs (Van Poelvoorde et al., 2020).

In recent years, NPs have emerged as new sources for development of antiviral drugs, with the potential to be developed into broad-spectrum antiviral drugs. The large number of compounds, comprehensive antiviral activity, and low cytotoxicity could be the advantages of using NPs as antiviral drugs (Mast et al., 2020). Most studies on antiviral agents have focused on the activity of NPs, which can inhibit different types of viruses. However, studies on related structural modifications and derivatization are relatively scarce, and the structure–activity relationship between NPs and their antiviral effects is seriously lacking, which can not well guide the synthesis of antiviral drugs derived from NPs.

This review surveyed NPs with antiviral activity and their derivatives in the past 2 decades, and summarized one hundred and twelve compounds’ structures and their antiviral activities. On this basis, we systematically explored the conformational relationships of different structural types of NPs in antiviral aspects. Alkaloids, quinones, flavonoids and terpenoids showed bright performance in exerting antiviral activity. Meanwhile, the derivatives of indole alkaloids, anthraquinones, naphthoquinones, flavonols and pentacyclic triterpenes could be substantially enhanced in their antiviral activities by appropriate structural modifications. These structural skeletons are very promising for the development of novel antiviral drugs and deserve further investigation. The positions 2, 3, and five on the indole ring were found to be important sites for antiviral activity in indole alkaloids. The introduction of amide and ester groups at the 2-position could enhance their antiviral activity, and the oxindole backbone formed by the introduction of carbonyl groups was also unique in antiviral. The number of hydroxyl groups in anthraquinones would correlate with their ability to exert antiviral activity. It was possible to enhance the inhibition of viruses by increasing the number of hydroxyl groups and keto-phenol systems on the same benzene ring. In addition, the introduction of epoxide structures in naphthoquinone compounds and the synthesis of naphthoquinone multimers could be employed for antiviral derivatization. The type and position of the substituent in the NP could have an effect on the antiviral activity of the compound. In some sites, the introduction of some groups would weaken the antiviral activity of natural products. For example, the introduction of 2′ hydroxyl groups on the B ring of flavonoids and 3-ketone groups in pentacyclic triterpenoids would have a detrimental effect upon antiviral activity. This knowledge could provide some ideas and directions for derivatization of the NP and strongly help to design and synthesize more antiviral drugs.

Drug resistance is a very challenging factor in the development of antiviral drugs. NPs offer great potential to combat this problem. Compared with drugs with single-spectrum antiviral activity, the multi-targeting of NPs could elicit more possibilities for antiviral agents. A combination of NPs with antiviral drugs could enhance the inhibitory and synergistic activity of antiviral drugs against drug-resistant strains. Artesunate (a derivative of artemisinin) has been shown to have activity against HCMV-susceptible, ganciclovir-resistant sublines, and clinical isolates without cross-resistance. Artesunate could offer a new approach to clinically refractory HCMV infections if standard antiviral therapies fail (Efferth et al., 2002; Schnepf et al., 2011). Studies have suggested that a combination of artesunate with the established antiviral drugs ganciclovir, cidofovir, maribavir, or phosphonate provide synergistic inhibition of the HCMV and reduce resistance to antiviral drugs (Drouot et al., 2016). Heredia and collaborators found that resveratrol increased the anti-HIV activity of tenofovir by 10-fold and restored susceptibility of TFV-resistant viruses. (Heredia et al., 2013). Kim’s team revealed that isoquercetin was highly effective in treatment of influenza viruses (even better than the positive control, amantadine). Moreover, isoquercetin could act in synergy with amantadine against influenza viruses and reduce resistance to amantadine (Kim et al., 2010). Haidari and his team discovered that a combination of pomegranate polyphenol extract and oseltamivir increased the anti-influenza effect of oseltamivir synergistically, and inhibited replication of the human influenza-A virus and H3N2 influenza virus *in vitro* (Haidari et al., 2009). Propolis is a non-toxic NP. Propolis and acyclovir have a strong synergistic effect against the herpes virus; perhaps a component of propolis affects cell division and increases the efficacy of acyclovir (Yildirim et al., 2016).

Current research on the actions of NPs against viruses has limitations. Many compounds have antiviral activity, but most of the active ingredients are present in low concentrations and difficult to isolate from NPs. Most studies have focused on the isolation and identification of active ingredients, but few studies have explored structural modifications. Many studies on antiviral activity showed only preliminary screening for antiviral activity and little research on the mechanisms and targets of NPs against viruses. Conducting clinical trials to demonstrate their efficacy and toxicity *in vivo* is not ethical, so most studies have been at the cellular level. This problem has restricted the development of antiviral drugs. Further research is required to assess the feasibility of NPs being used as antiviral drugs in clinical practice. It has been proposed that the antiviral activity of NPs could be deepened through a combination of technologies, such as high-throughput screening, synthetic biology, metabolic engineering, and medicinal chemistry. In recent years, artificial intelligence has been applied gradually for the discovery and development of drugs. Computer-aided drug design as well as artificial intelligence drug discovery and design have started to become the core technologies for innovative drug research because they have a short development cycle and high hit rate. These technologies could provide a new impetus to develop safe and efficacious antiviral drugs faster, and drive the development of innovative drugs.

## Abbreviations

Adv, adenovirus; AS, Acetylshikonin; Atg12, autophagy-related gene 12; Atg5, autophagy-related gene five; CA, Caffeic acid; CC_50_, cytotoxic concentration of extracts to cause death to 50% of viable cells; CCR5, C-C chemokine receptor five; CDV, Canine distemper virus; CHA, Chlorogenic acid; CHB, chronic HBV; CHIKV, Chikungunya virus; COVID-19, Coronavirus disease 2019; CVA16, coxsackievirus A16; CVB3, coxsackievirus B3; DENV, Dengue virus; EBV, Epstein-Barr virus; EC_50_, half-maximal effective concentration; ED_50_, median effective dose; EV71, enterovirus 71; GA, Glycyrrhetinic acid; GaLA, galacturonic acid; GL, Glycyrrhizin; H3N2, influenza virus A3/Beijing/30/95; HBeAg, hepatitis B virus e antigen; HBsAg, hepatitis B virus surface antigen; HBV, hepatitis B virus; HBx, HBV X; HCMV, Human cytomegalo vrius; HCoV-NL63, human coronavirus NL63; HCV, hepatitis C virus; HIV, human immunodeficiency virus; HIV-1RT, HIV-1 reverse transcriptase; HSV, herpes simplex viruses; IAV, influenza A virus; IC_50_, half-maximal inhibitory concentration; IFN-α, interferon-α; Keap1/Nrf2, Kelch-like ECH-associated protein 1/Nuclear factor (erythroid-derived 2)-like two; LC3-II, autophagy-related protein light chain three; MDCK, Madin-Darby canine kidney; NNRTI, non-nucleoside reverse transcriptase inhibitor; NPs, Natural products; OA, Oleanolic acid; SARS-CoV-2, severe acute respiratory syndrome-coronavirus two; SFTSV, severe fever with thrombocytopenia syndrome virus; SI, selectivity index; TMV, tobacco mosaic virus; UA, Ursolic acid; ZIKV, Zikavirus.
