# Supplementary material for: Research progress on the antiviral activities of natural products and their derivatives: Structure–activity relationships
Source: Front Chem. 2022 Oct 12;10:1005360. doi: 10.3389/fchem.2022.1005360 (PMC9596788; doi:10.3389/fchem.2022.1005360)
Supplement: Supplementary file 1 [file Table1.pdf]

Table 1 Natural products and their derivatives with antiviral activity

Symbol	Type	Compound	Structure	Targets and effects	Source	Ref.
1A	Alkaloid	Isatigotindolediosides C	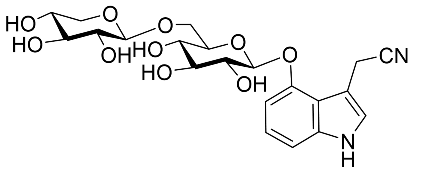	Anti-coxsackie-B3 (IC_{50} = 33.3 μM)	*Isatis indigotica* roots	(Meng, et al. 2017b)
2A		Isatigotindolediosides E	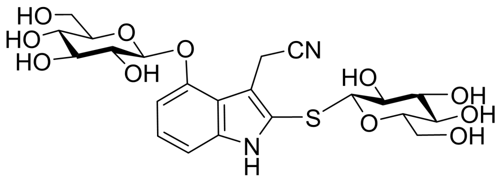	Anti-coxsackie-B3 (IC_{50} = 33.3 μM)	*Isatis indigotica* roots	(Meng et al., 2017b)
3A		Calanthoside	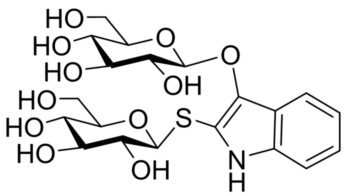	Anti-coxsackie-B3 (IC_{50} = 33.3 μM)	*Isatis indigotica* roots green seaweed *Gayralia oxysperma*	(Meng et al., 2017b)
4A		Isatibisindosulfonic acid B	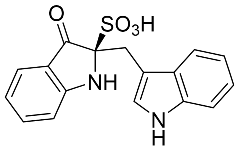	Anti-coxsackie-B3 (IC_{50} = 33.3 μM)	*Isatis indigotica* roots	(Meng, et al. 2017a)
5A		Isatindosulfonic acid B.	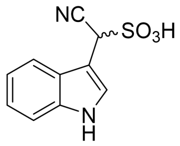	Anti-coxsackie-B3 (IC_{50} = 33.3 μM)	*Isatis indigotica* roots	(Meng, et al. 2017a)

Symbol	Type	Compound	Structure	Targets and effects	Source	Ref.
6A		2-(3-cyanomethyl-4-methoxy-1*H*-indol-7-yl)-2-(1*H*-indol-3-yl)acetonitrile	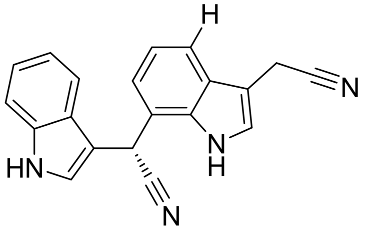	Anti-coxsackie-B3 (IC_{50} = 3.70−12.35 μM)	*Isatis indigotica* roots	(Chen et al., 2012)
7A		(−)-(R)-2-(3-cyanomethyl-4-methoxy-1*H*-indol-7-yl)-2-(4-methoxy-1*H*-indol-3-yl)acetonitrile	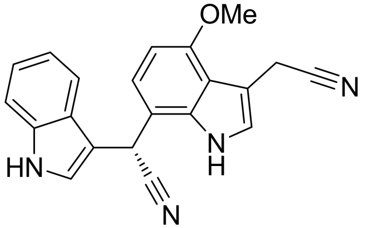	Anti-Anti-H3N2 (IC_{50} = 3.70−12.35 μM)	*Isatis indigotica* roots	(Chen et al., 2012)
8A	Alkaloid	(+)-(S)-2-{7-[1-(4-hydroxyphenyl)-ethyl]-4-me-thoxy-1*H*-indol-3-yl}acetonitrile	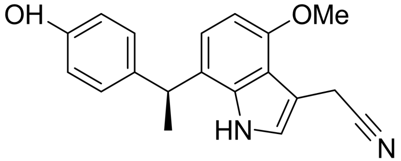	Anti-Anti-H3N2 (IC_{50} = 3.70−12.35 μM)	*Isatis indigotica* roots	(Chen et al., 2012)
9A		arvelexin	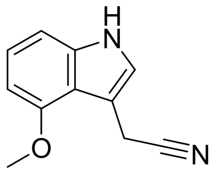	Anti-Anti-H3N2 (IC_{50} = 3.70−12.35 μM)	*Isatis indigotica* roots	(Chen et al., 2012)
10A		(±)-2-(2-Oxoindolin-3-yl)acetamide	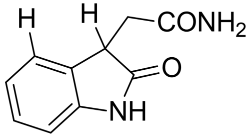	Anti-coxsackie-B3 (IC_{50} = 6.87 μM)	*Isatis indigotica* roots	(Chen et al., 2012)

Symbol	Type	Compound	Structure	Targets and effects	Source	Ref.
11A	Alkaloid	17-nor-excelsinidine	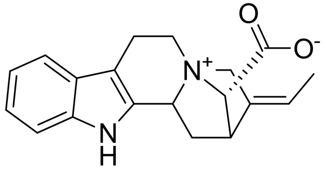	Anti-Adv (EC_{50} = 0.94 µg/mL) Anti-HSV (EC_{50} = 1.09 µg/mL)	*Alstonia scholaris*	(Zhang et al., 2014)
12A		Caulerpin	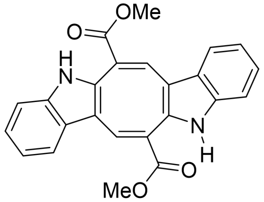	Anti-CHIKV (EC_{50} = 0.8 µg/mL) Anti-HSV-1 (EC_{50} = 1.29 µM)	Marine green alga *Caulerpa racemosa*	(Esteves et al., 2019) (Macedo et al., 2012)
13A		Forsyqinlingine A	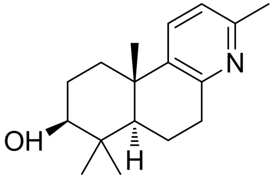	Anti-H1N1 (IC_{50} = 6.9 µM) Anti-RSV (IC_{50} = 5.0 µM)	*Forsythia suspensa*	(Li et al., 2021)
14A		Forsyqinlingine B	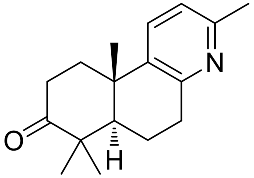	Anti-H1N1 (IC_{50} = 7.7 µM) Anti-RSV (IC_{50} = 4.8 µM)	*Forsythia suspensa*	(Li et al., 2021)
15A		Forsyqinlingine C	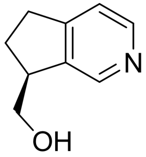	Anti-H1N1 (IC_{50} = 11.9 µM) Anti-RSV (IC_{50} = 13.5 µM)	*Forsythia suspensa*	(Li et al., 2022)

Symbol	Type	Compound	Structure	Targets and effects	Source	Ref.
16A	Alkaloid	Forsyqinlingine D	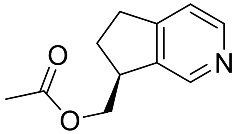	Anti-H1N1 (IC_{50} = 15.1 μM) Anti-RSV (IC_{50} = 14.0 μM)	*Forsythia suspensa*	(Li et al., 2022)
17A		Lonijaposide O	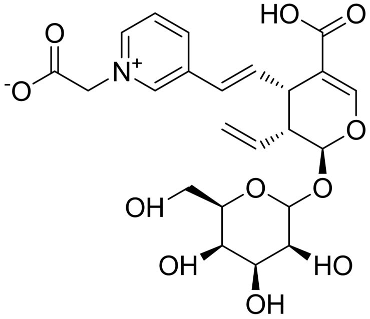	Anti-H3N2 (IC_{50} = 11.6 μM)	Flower buds of Lonicera japonica	(Yu et al., 2013)
18A		Lonijaposide R	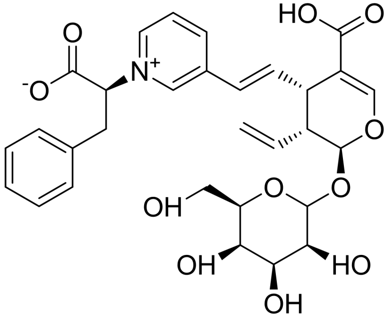	Anti-H3N2 (IC_{50} = 6.8 μM) Anti-coxsackie-B3 (IC_{50} = 12.3 μM)	Flower buds of *Lonicera japonica*	(Yu et al., 2013)

Symbol	Type	Compound	Structure	Targets and effects	Source	Ref.
19A	Alkaloid	Lonijaposide T	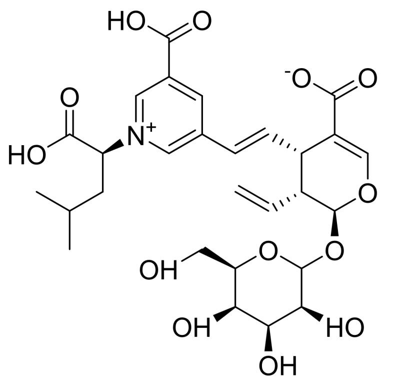	Anti-H3N2 influenza virus (IC_{50} = 10.3 μM)	Flower buds of *Lonicera japonica*	(Yu et al., 2013)
20A		Lonijaposide W	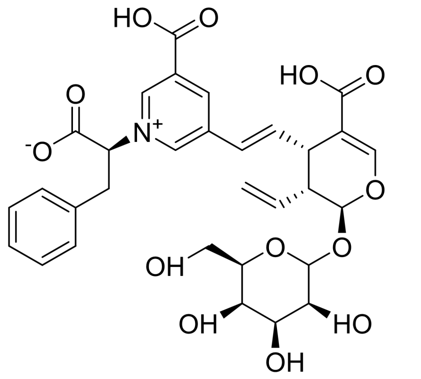	Anti-H3N2 influenza virus (IC_{50} = 8.2 μM)	Flower buds of *Lonicera japonica*	(Yu et al., 2013)
21A		(−)-cytisine	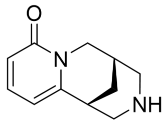	Anti-influenza virus (ED_{50} = 109 μg/mL)	*Spalathus linearis*	(Tsypysheva et al., 2013)

Symbol	Type	Compound	Structure	Targets and effects	Source	Ref.
22A	Alkaloid	11-(3-bromobenzoyl)-7,11-diazatricyclo[7.3.1.0^{2,7}]trideca-2,4-dien-6-one	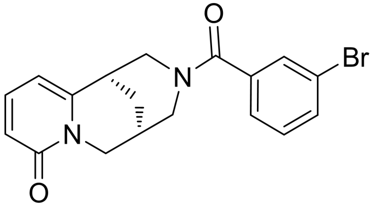	Anti-influenza virus (ED_{50} = 44 μg/mL)	*Spalathus linearis*	(Tsypysheva et al., 2013)
23A		*N*-(12-Methylcytisin-3-yl)-*N'*-phenylurea	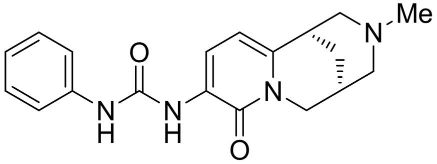	Anti-influenza virus (ED_{50} = 57 μg/mL)	*Spalathus linearis*	(Tsypysheva et al., 2013)
24A		Warifteine	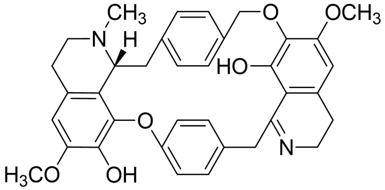	Anti-ZIKV (IC_{50} = 2.2 μg/mL) Anti-Dengue virus (IC_{50} = 2.00 μg/mL)	*Cissampelos sympodialis* rhizomes	(da Silva et al., 2021a) (da Silva et al., 2021b)
25A		Methylwarifteine	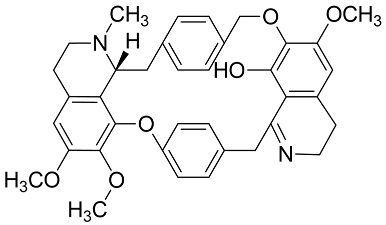	Anti-ZIKV (IC_{50} = 3.5 μg/mL)	*Cissampelos sympodialis* rhizomes	(da Silva et al., 2021b)
26A		Dehydrocheilanthifoline	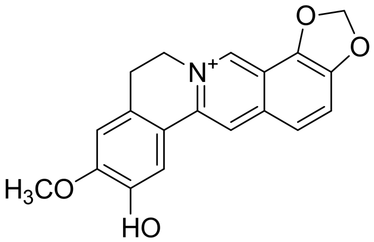	Anti-HBsAg (IC_{50} = 15.84 μM) Anti-HBeAg (IC_{50} = 17.12 μM)	*Coptidis Rhizoma*	(Zeng et al., 2013)

Symbol	Type	Compound	Structure	Targets and effects	Source	Ref.
27A	Alkaloid	(+)-12α-hydroxysophocarpine	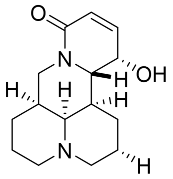	Anti-H3N2 (IC$_{50}$ = 84.70 μM)	Rhizomes of *Sophora tonkinensis*	(Pan et al., 2015)
28A		(−)-12β-hydroxy-sophocarpine	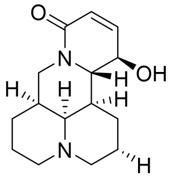	Anti-H3N2 (IC$_{50}$ = 242.46 μM)	Rhizomes of *Sophora tonkinensis*	(Pan et al., 2015)
29A		(+)-sophoramine	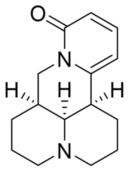	Anti-H3N2 (IC$_{50}$ = 63.07 μM)	Rhizomes of *Sophora tonkinensis*	(Pan et al., 2015)
30A		(−)-12β-hydroxyoxy-sophocarpine	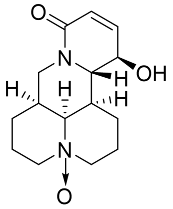	Anti-coxsackie-B3 (IC$_{50}$ = 26.62 μM)	Rhizomes of *Sophora tonkinensis*	(Pan et al., 2015)
31A		(−)-9α-hydroxysophocarpine	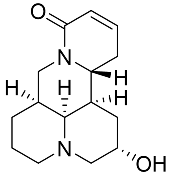	Anti-coxsackie-B3 (IC$_{50}$ = 197.22 μM)	Rhizomes of *Sophora tonkinensis*	(Pan et al., 2015)

Symbol	Type	Compound	Structure	Targets and effects	Source	Ref.
32A	Alkaloid	(+)-sophoranol	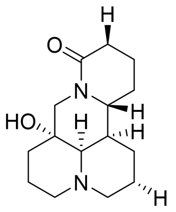	Anti-coxsackie-B3 (IC_{50} = 252.18 μM)	Rhizomes of *Sophora tonkinensis*	(Pan et al., 2015)
33A		(−)-14β-hydroxymatrine	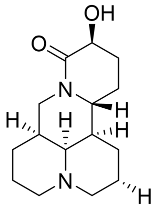	Anti-coxsackie-B3 (IC_{50} = 184.14 μM)	Rhizomes of *Sophora tonkinensis*	(Pan et al., 2015)
34A		Tylophorine B	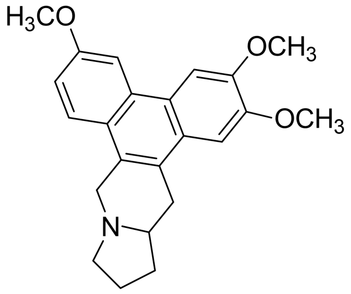	Anti-TMV (IC_{50} = 2.4 nM)	*Cynanchum komarovii*	(Xi et al., 2006)
35A		Anisomycin	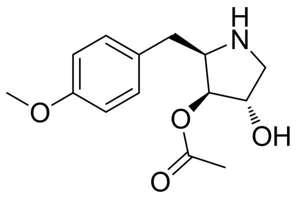	Anti-ZIKV (IC_{50} = 7.9 ± 1.2 nM) Anti-DENV-2 (IC_{50} = 32.2±1.1 nM) Anti-SARS-CoV-2 (IC_{50} = 31.4 nM)	*Botrytis cinerea*	(Quintana et al., 2020) (Huang et al., 2020)

Symbol	Type	Compound	Structure	Targets and effects	Source	Ref.
36A	Alkaloid	Piperine	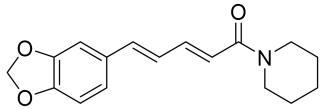	Anti-HBsA (IC_{50} = 0.15 mM) Anti-HBeAg (IC_{50} = 0.14 mM)	*Piper nigrum*	(Jiang et al., 2013)
37A		5-Bromo-3-[(3,5-dimethyl-phenyl)sulfonyl]-N-(pyrrolidin-1-ylmethyl)-1*H*-indole-2-carboxamide	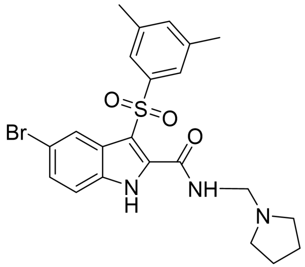	Anti-HIV-1 (IC_{50} = 1.3 nM)	Derivatives	(La Regina et al., 2011)
38A		5-Chloro-3-[[3-[(*E*)-2-cyanovinyl]-5-methyl-phenyl]-methoxy-phosphoryl]-1*H*-indole-2-carboxamide (IDX899)	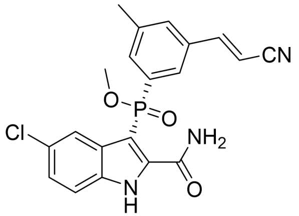	Anti-HIV-1 (IC_{50} = 0.34 μM)	Derivatives	(Dousson et al., 2016)
39A		ethyl 5-bromo-3-(1-phenylcyclopropyl)-1*H*-indole-2-carboxylate	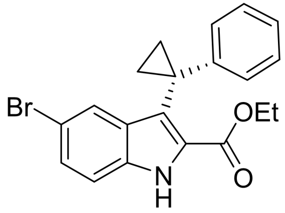	Anti-HIV-1 (IC_{50} = 0.066 μM)	Derivatives	(Hassam et al., 2012)

Symbol	Type	Compound	Structure	Targets and effects	Source	Ref.
40A	Alkaloid	5-chloro-3-(2-(4-fluorophenyl)-2-oxoethyl)-3-hydroxyindolin-2-one	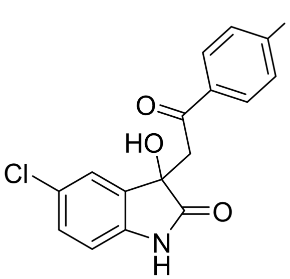	Anti-HIV-1 (IC_{50} = 5.92 μM)	Derivatives	(Chander et al., 2018)
41A		5-bromo-3-hydroxy-3-(2-oxo-2-(m-tolyl)ethyl)indolin-2-one	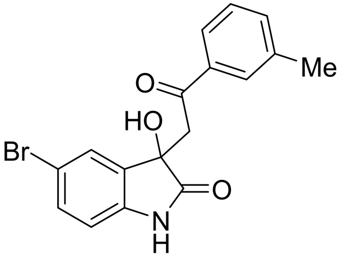	Anti-HIV-1 (IC_{50} = 1.38 μM)	Derivatives	(Chander et al., 2018)
42A		5-bromo-3-hydroxy-3-(2-(2-methoxyphenyl)-2-oxoethyl)indolin-2-one	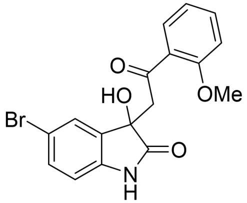	Anti-HIV-1 (IC_{50} = 0.82 μM)	Derivatives	(Chander et al., 2018)
43A		5-bromo-3-(2-(2-chlorophenyl)-2-oxoethyl)-3-hydroxyindolin-2-one	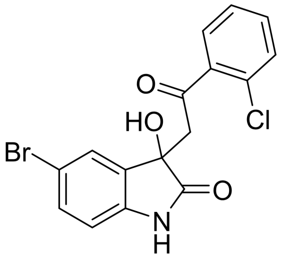	Anti-HIV-1 (IC_{50} = 2.03 μM)	Derivatives	(Chander et al., 2018)

Symbol	Type	Compound	Structure	Targets and effects	Source	Ref.
44A	Alkaloid	5-chloro-3-(2-(2-chlorophenyl)-2-oxoethyl)-3-hydroxyindolin-2-one	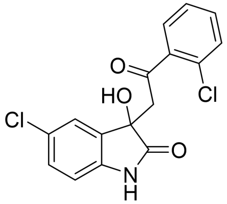	Anti-HIV-1 (IC_{50} = 0.76 μM)	Derivatives	(Chander et al., 2018)
45A		5-chloro-3-(2-(3-chlorophenyl)-2-oxoethyl)-3-hydroxyindolin-2-one	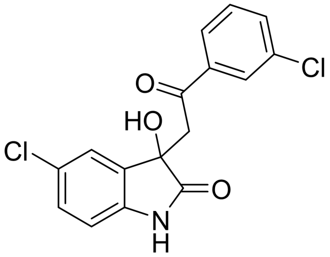	Anti-HIV-1 (IC_{50} = 34.25 μM)	Derivatives	(Chander et al., 2018)
46A		5-chloro-3-(2-(3,4-dichlorophenyl)-2-oxoethyl)-3-hydroxyindolin-2-one	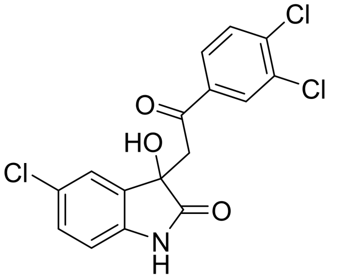	Anti-HIV-1 (IC_{50} = 68.86 μM)	Derivatives	(Chander et al., 2018)
1Q	Quinones	Zeylanone epoxide	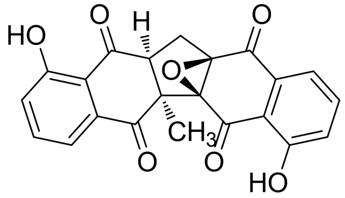	Anti H1N1 influenza virus (IC_{50} = 0.65 ± 0.01 μM) Anti H3N2 influenza virus (IC_{50} = 1.6 ± 0.09 μM)	*Diospyros anisandra*	(Cetina-Montejo et al., 2019)

Symbol	Type	Compound	Structure	Targets and effects	Source	Ref.
2Q	Quinones	Acetylshikonin (AS)	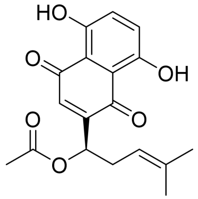	Anti-CVA16 (EC_{50} = 0.04μM)	*Arnebiae Radix*	(Liu et al., 2019b)
3Q		Dicoumarol	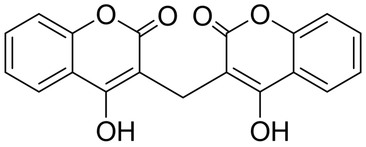	Anti-CHB (EC_{50} = 100 μM)	*Trifolium*	(Cheng et al., 2021b)
4Q		Aloe-emodin	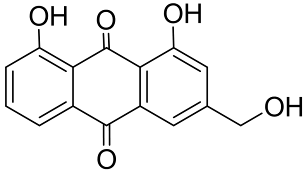	Anti-HBV (Inhibition: 83%)	*Rhei Radix et Rhizoma*	(Parvez et al., 2019)
5Q		Droserone	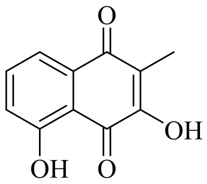	Anti-H1N1 (IC_{50} > 100 μM)	Derivatives	(Cetina-Montejo et al., 2019)
6Q		Plumbagin	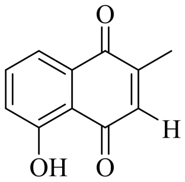	Anti-H1N1 (IC_{50} > 6.25 μM)	Derivatives	(Cetina-Montejo et al., 2019)

Symbol	Type	Compound	Structure	Targets and effects	Source	Ref.
7Q	Quinones	2,3-epoxiplumbagin	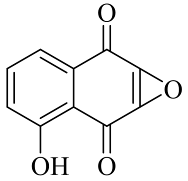	Anti-H1N1 (IC_{50} > 12.5 μM)	Derivatives	(Cetina-Montejo et al., 2019)
8Q		3,3-biplumbagin	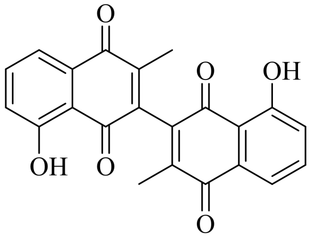	Anti-H1N1 (IC_{50} > 0.78 μM)	Derivatives	(Cetina-Montejo et al., 2019)
9Q		1,4-dihydroxyanthraquinone	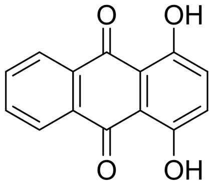	Anti-HCV (IC_{50} = 54 μM)	Derivatives	(Furuta et al., 2015)
10Q		1,2-dihydroxyanthraquinone	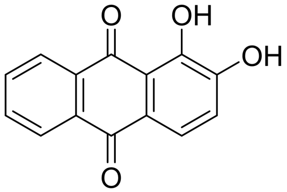	Anti-HCV (IC_{50} > 200μM)	Derivatives	(Furuta et al., 2015)
11Q		1,5-dihydroxyanthraquinone	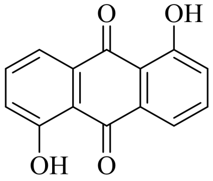	Anti-HCV (IC_{50} > 200 μM)	Derivatives	(Furuta et al., 2015)

Symbol	Type	Compound	Structure	Targets and effects	Source	Ref.
12Q	Quinones	1,8-dihydroxyanthraquinone	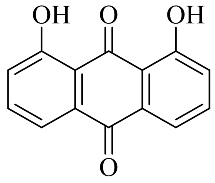	Anti-HCV ($IC_{50} > 200$ μM)	Derivatives	(Furuta et al., 2015)
13Q		1,4,5,8-tetrahydroxyanthraquinone	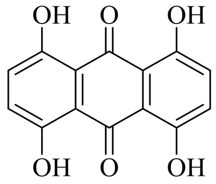	Anti-HCV ($IC_{50} = 6$ μM)	Derivatives	(Frecentese et al., 2016)
14Q		1,2,3-trihydroxyanthraquinone	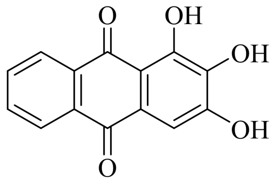	Anti-HCV ($IC_{50} = 18$ μM)	Derivatives	(Frecentese et al., 2016)
15Q		1,2,4-trihydroxyanthraquinone	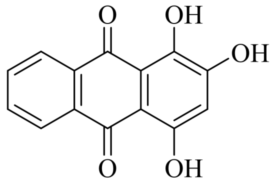	Anti-HCV ($IC_{50} = 11$ μM)	Derivatives	(Frecentese et al., 2016)

Symbol	Type	Compound	Structure	Targets and effects	Source	Ref.
16Q	Quinones	Hypericin	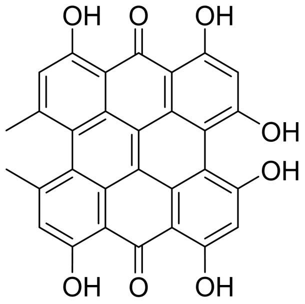	Anti-HCV (IC_{50} = 3μM)	*Hypericum perforatum L.*	(Furuta et al., 2015)
17Q		Sennidin A	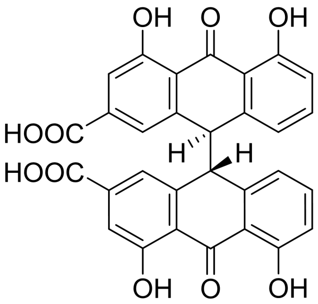	Anti-HCV (IC_{50} = 0.8 μM)	*Folium sennae*	(Furuta et al., 2015)
18Q		*N,N′*-(2,2′-(9,10-Dioxo-9,10-dihydroanthracene-2,7-diyl)bis(azanediyl)bis(2-oxo-ethane-2,1-diyl))bis(2,6-diaminohexanamide)-tetra-trifluoroacetate	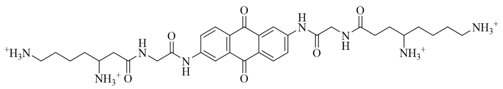	Anti-HIV-1 (IC_{50} = 2.12 ± 0.16 μM)	Derivatives	(Frecentese, et al. 2016)
1F	Flavonoid	Quercetin	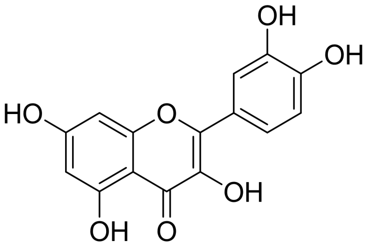	Anti-CDV (IC_{50} = 11.72 ± 0.85 μg/mL) Anti-HIV (IC_{50} = 88.98 μM) Anti H1N1 influenza virus (ED_{50} = 48 μM)	Widespread in nature	(Carvalho et al., 2013a) (Pasetto et al., 2014) (Thapa et al., 2012)

Symbol	Type	Compound	Structure	Targets and effects	Source	Ref.
2F	Flavonoid	Rutin	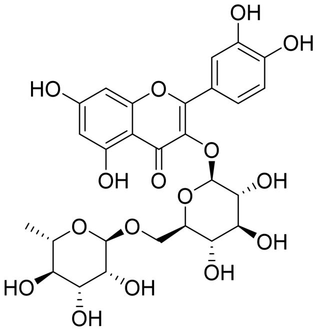	Anti-CDV (IC_{50} = 10.41 ± 0.12 μg/mL)	*Sophora japonica L.*	(Carvalho et al., 2013a)
3F		Isoquercetin	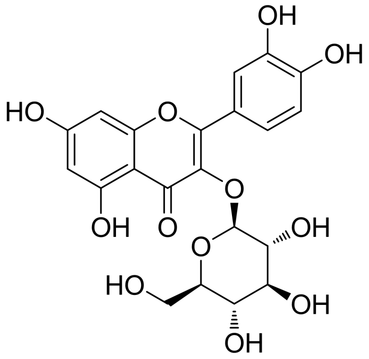	Anti-H1N1 influenza virus (ED_{50} = 1.2 μM)	Widespread in nature	(Kim et al., 2010)
4F		Baicalin	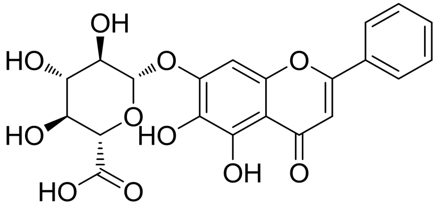	Anti-CHIKV (EC_{50} = 7 μM) Anti-DENV-2 (IC_{50} = 13.50 μg/mL)	*Scutellaria baicalensis Georgi*	(Oo et al., 2018) (Moghaddam et al., 2014)
5F		Baicalein	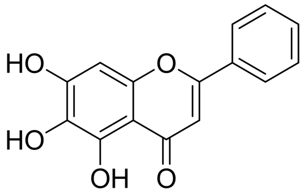	Anti-CHIKV (IC_{50} = 1.891 μg/mL) Anti-DENV-2 (IC_{50} = 6.46 μg/mL)	*Scutellaria baicalensis Georgi*	(Lani et al., 2016) (Moghaddam et al., 2014)

Symbol	Type	Compound	Structure	Targets and effects	Source	Ref.
6F	Flavonoid	Hesperidin	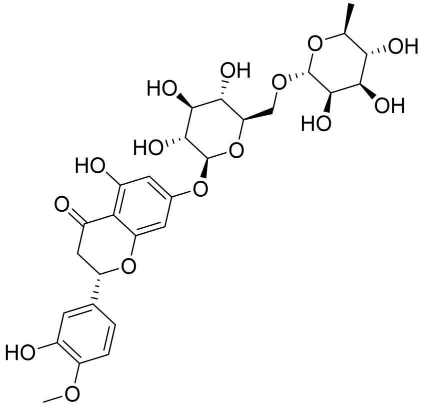	Anti-CDV<br>(IC_{50} = 11.09 ± 0.45 μg/mL)	*Citrus reticulata Blanco*	(Carvalho et al., 2013a)
7F		Epigallocatechin-3-gallate (EGCG)	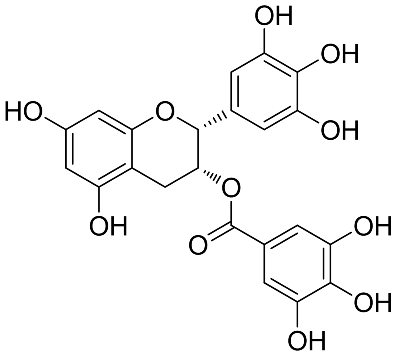	Anti-HBV<br>(HBsAg inhibition: 53%, HBeAg inhibition: 44%)<br>Anti-H1N1 influenza virus<br>(ED_{50} = 8.3 μM)	*Camellia sinensis*	(Pang et al., 2014)<br>(Thapa et al., 2012)
8F		Myricetin	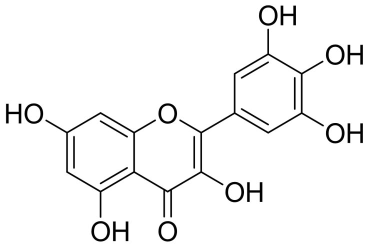	Anti-HIV<br>(IC_{50} = 20.43 μM)	*Myrica rubra*	(Pasetto, et al. 2014)

Symbol	Type	Compound	Structure	Targets and effects	Source	Ref.
9F	Flavonoid	Pinocembrin	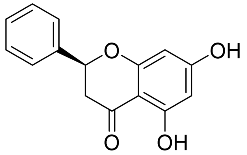	Anti-HIV (IC_{50} = 346.75 μM)	*Colla Apis*	(Pasetto, et al. 2014)
10F		Morin	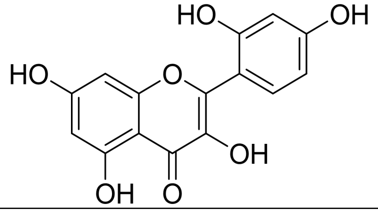	Anti-CDV (IC_{50} = 34.02 μg/mL)	*Phellinus igniarius*	(Carvalho et al., 2013a)
11F		Quercetin-3-gallate	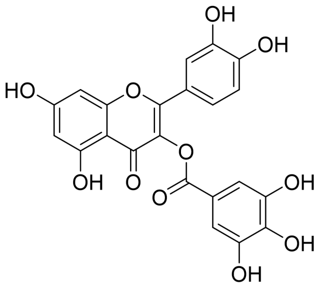	Anti-H1N1 influenza virus (ED_{50} = 9.1 μM)	Widespread in nature	(Thapa, et al. 2012)
12F		2-(3,4-Dihydroxyphenyl)-5,7-dihydroxy-4-oxo-4*H*-chromen-3-yl 2,5-dihydroxybenzoate	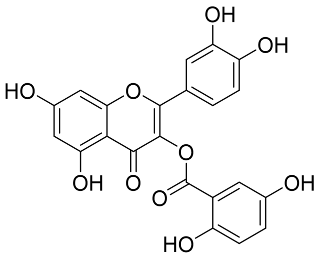	Anti-H1N1 influenza virus (ED_{50} = 19.4 μM)	Widespread in nature	(Thapa, et al. 2012)

Symbol	Type	Compound	Structure	Targets and effects	Source	Ref.
13F	Flavonoid	5,7-Dihydroxy-2-(3,4-dihydroxyphenyl)-4-oxo-4H-chromen-3-yl 4-hydroxy-3-aminobenzoate	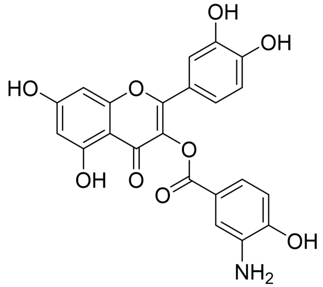	Anti-H1N1 influenza virus (ED_{50} = 22.6 μM)	Widespread in nature	(Thapa, et al. 2012)
14F		2-(3,4-Dihydroxyphenyl)-5,7-dihydroxy-4-oxo-4H-chromen-3-yl 4-amino-3-hydroxybenzoate	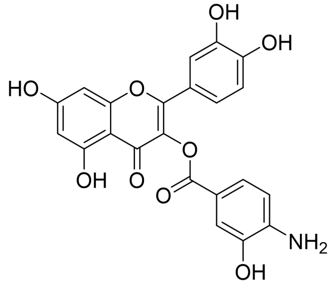	Anti-H1N1 influenza virus (ED_{50} = 24.1 μM)	Widespread in nature	(Thapa, et al. 2012)
15F		2-Hydroxy-5-(3,5,7-trihydroxy-4-oxo-4H-chromen-2-yl)phenyl 3,4,5-trihydroxybenzoate	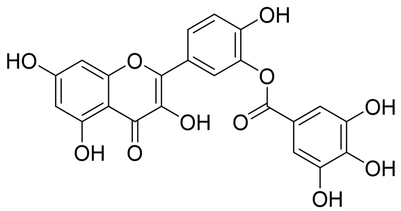	Anti H1N1 (ED_{50} > 50 μM)	Derivatives	(Thapa, et al. 2012)
16F		3,5,7-(Trihydroxy)-2-[4-hydroxy-3-(3-aminopropyloxy)phenyl]-4-oxo-4*H*-chromene	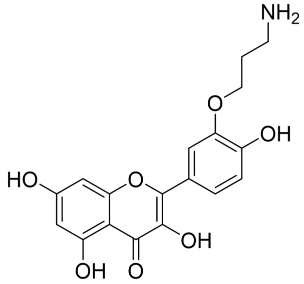	Anti H1N1 (ED_{50} > 50 μM)	Derivatives	(Thapa, et al. 2012)

Symbol	Type	Compound	Structure	Targets and effects	Source	Ref.
17F	Flavonoid	3,7-(Dihydroxy)-2-(3,4-dihydroxyphenyl)-5-propyloxy-4-oxo-4*H*-chromene	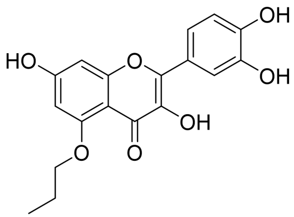	Anti H1N1 (ED_{50} > 50 μM)	Derivatives	(Thapa, et al. 2012)
1T	Terpene	Artemisinin	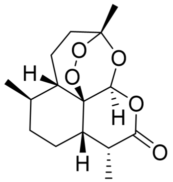	Anti-HBV (IC_{50} = 55 μmol/L)	*Artemisia annua*	(Wohlfarth and Efferth, 2009)
2T		Artesunate	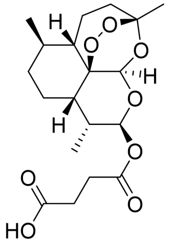	Anti-HBV (IC_{50} = 2.3 μmol/L)	Derivatives	(Wohlfarth and Efferth, 2009)
3T		(1R,3aS,5aR,5bR,9S,11aR)-5a,5b,8,8,11a-pentamethyl-9-((4-oxo-4-(((3R,9R,10S,12R,12aR)-3,6,9-trimethyldecahydro-12*H*-3,12-epoxy[1,2]dioxepino[4,3-i]isochromen-10-yl)oxy)butanoyl)oxy)-1-(prop-1-en-2-yl)icosahydro-3aH-cyclopenta[a]chrysene-3a-carboxylic acid	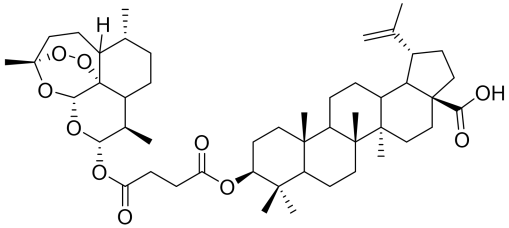	Anti-HCMV (EC_{50} = 0.24 μM)	Derivatives	(Karagoz et al., 2019)

Symbol	Type	Compound	Structure	Targets and effects	Source	Ref.
4T	Terpene	Andrographolide	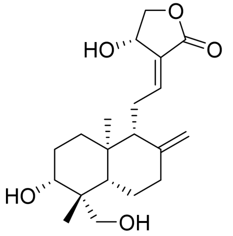	Anti-DENV-2 (EC_{50} = 21.304 μM in Hep G2 cells) (EC_{50} = 22.739 μM in HeLa cells)	*Andrographis paniculata*	(Panraksa et al., 2017)
5T		Phomanolide	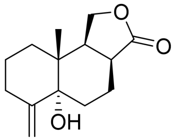	Anti-H1N1 influenza virus (IC_{50} = 2.96 ± 0.64 μg/mL)	*Phoma* species isolated from the roots of *Aconitum vilmorinianum.*	(Liu et al., 2019a)
6T		Xiamycin	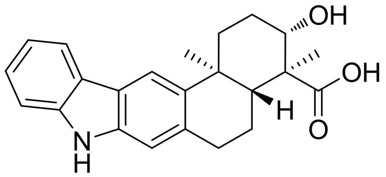	Anti-HIV (IC_{50} > 30 μM)	Streptomyces species GT2002/1503	(Ding et al., 2010)
7T		Celastrol	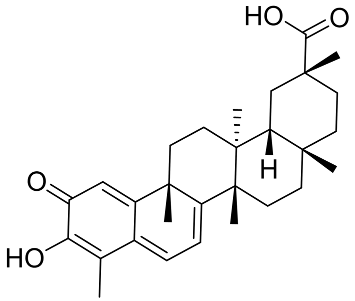	Anti-HCV (EC_{50} = 0.37 ± 0.022 μM)	Root extracts of *Tripterygium wilfordii*	(Tseng et al., 2017)

Symbol	Type	Compound	Structure	Targets and effects	Source	Ref.
8T	Terpene	3,16-Dioxo-*N*-(2,3,4,6-tetra-*O*-acetyl-β-D-galactopyranosyl)olean-12-en-28-amide	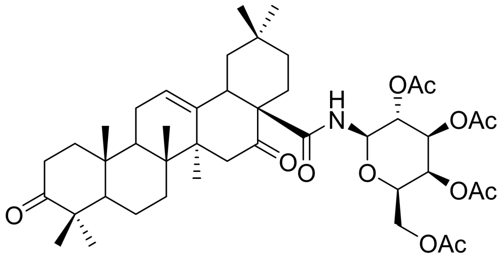	Anti-EBOV (IC_{50} = 59.2 ± 1.6 nM)	Derivatives	(Si et al., 2018)
9T		Glycyrrhizin (GL)	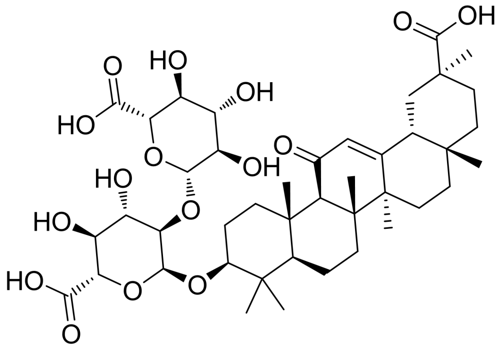	Anti-HCV (EC_{50} = 16.5 μM)	Licorice *(Glycyrrhizae glabra)* root	(Matsumoto et al., 2013)
10T		3-O-(3‘,3‘-dimethylsuccinyl)-oleanolic acid	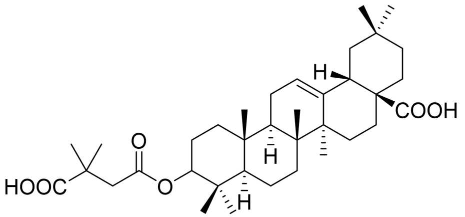	Anti-HIV-1 (EC_{50} = 0.32 μM)	Derivatives	(Yu et al., 2006)

Symbol	Type	Compound	Structure	Targets and effects	Source	Ref.
11T	Terpene	3, 11-Dioxo-18β-olean-12-en-30-oic acid	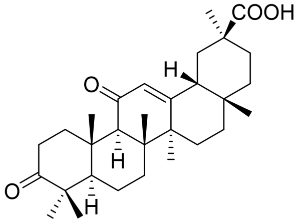	Anti-HBV (IC_{50} = 432.54 μM)	Derivatives	(Wang et al., 2012a)
12T		Glycyrrhetinic acid (GA)	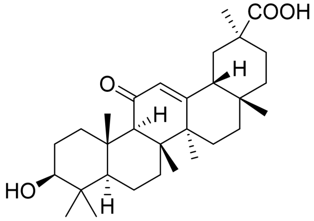	Anti-HBV (IC_{50} = 20.86 μM)	Licorice *(Glycyrrhizae glabra)* root	(Wang et al., 2012a)
13T		3α-O-[2,4-Di-O-(α-L-rhamnopyranosyl)-β-D-glucopyranosyl]-12-en-28-oic acid methyl ester	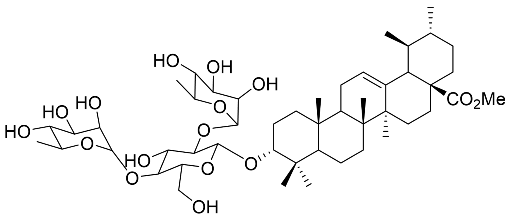	Anti-H5N1 (IC_{50} = 1.33 ± 0.15 μM)	Derivatives	(Song et al., 2015)
14T		methyl ursolate 3-O-b-chaco-trioside	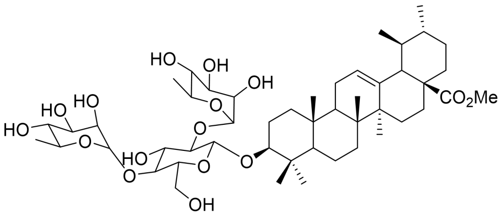	Anti-H5N1 (IC_{50} = 1.02 ± 0.13 μM)	Derivatives	(Song et al., 2015)

Symbol	Type	Compound	Structure	Targets and effects	Source	Ref.
15T	Terpene	Ursolic acid (UA)	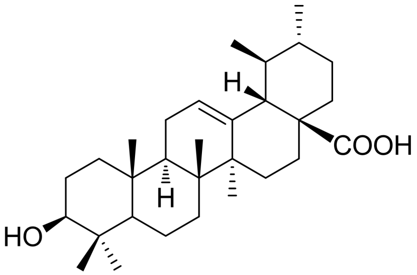	Anti-HCV (IC_{50} = 10.6 μg/ml)	*Fructus Ligustri Lucidi*	(Kong et al., 2013)
16T		Oleanolic acid (OA)	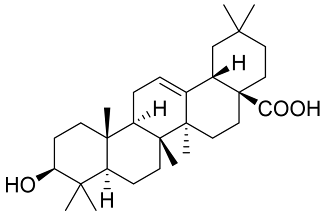	Anti-HCV (IC_{50} = 2.9 μg/ml)	*Fructus Ligustri Lucidi*	(Kong et al., 2013)
17T		4-Hydroxy-3, 4-seco-olean-12-en-28-oic acid 3, 4 lactone	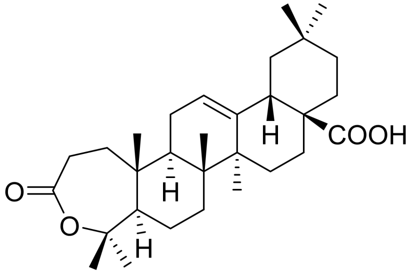	Anti-HBV (IC_{50} = 0.86 μM)	Derivatives	(Li et al., 2018)
18T		Olean-12α-hydroxy-3-ε-lacton-28-oic acid γ-lactone	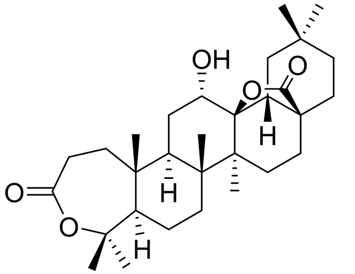	Anti-HBV (IC_{50} = 149.1 μM)	Derivatives	(Li et al., 2018)

Symbol	Type	Compound	Structure	Targets and effects	Source	Ref.
1R	Organic acid	Cis-cinnamic acid	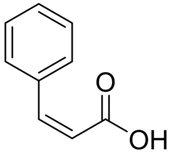	Anti-CDV (IC_{50} = 4.46 ± 0.78 µg/mL)	*Cinnamomum cassia Presl*	(Carvalho et al., 2013a)
2R		Ferulic acid	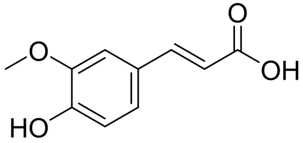	Anti-CDV (IC_{50} = 3.56 ± 0.13 µg/mL)	*Ferula assafoetida L.*	(Carvalho, et al. 2013)
3R		*Trans*-cinnamic acid	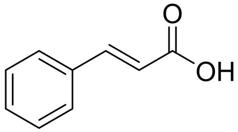	Anti-CDV (IC_{50} = 23.11 ± 4.48 µg/mL)	*Cinnamomum cassia Presl*	(Carvalho et al., 2013a)
4R		Chlorogenic acid (CHA)	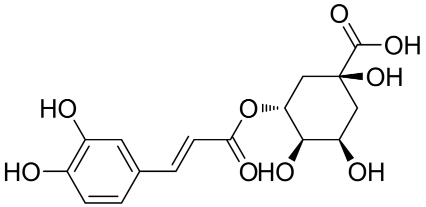	Anti-H1N1 (EC_{50} = 44.87 µM) Anti-H3N2 (EC_{50} = 62.33 µM) Anti-HCoV-NL63 (IC_{50} = 43.45 µM)	*Lonicera japonica Thunb*	(Ding, et al. 2017) (Weng, et al. 2019)
5R		Caffeic acid (CA)	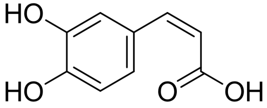	Anti-HCoV-NL63 (IC_{50} = 3.54 µM) Anti-HCV (IC_{50} = 100 ± 20 µM) Anti-SFTSV (IC_{50} = 0.048 mM)	*Lonicera japonica Thunb*	(Weng et al., 2019) (Shen et al., 2018) (Ogawa et al., 2018)

Symbol	Type	Compound	Structure	Targets and effects	Source	Ref.
1O	Others	Tatanan A	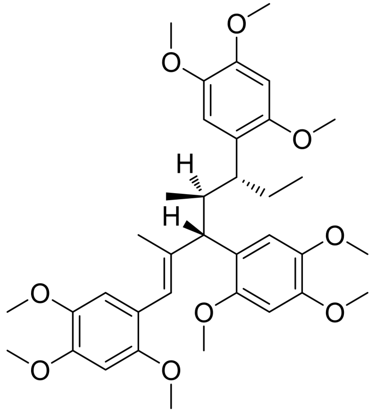	Anti-DENV-2 (EC_{50} = 3.9 μM)	*Acorus calamus L.* roots	(Yao, et al. 2018)
2O		Manassantin B	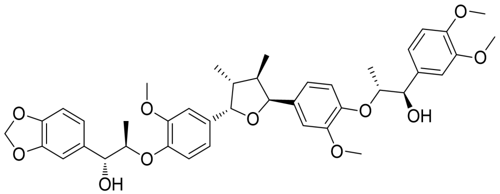	Anti-EBV (EC_{50} = 1.72 μM)	*Saururus chinensis* roots	(Cui, et al. 2014)
3O		Lutein	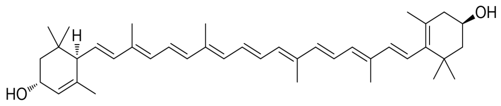	Anti-HBV (HBsAg < 60%)	Widespread in nature	(Pang, et al. 2010)
4O		Pyropheophorbide	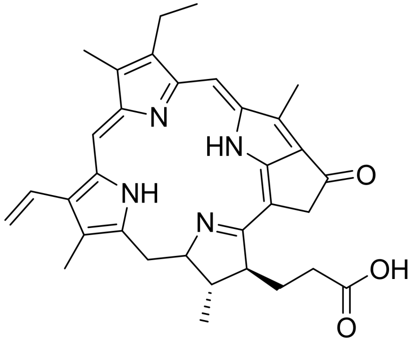	Anti-HCV (IC_{50} = 0.2 μg/mL)	*M. citrifolia*	(Ratnoglik et al., 2014)

Symbol	Type	Compound	Structure	Targets and effects	Source	Ref.
5O	Others	Diosgenin (3β-hydroxy-5-spirostene)	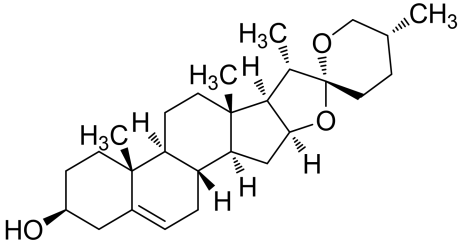	Anti-HCV (EC_{50} = 3.8 μM)	*Dioscorea zingiberensis C. H. Wright*	(Wang et al., 2011b)
1P		Arabinoxylan	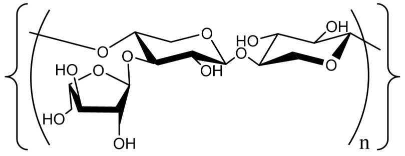	Anti-HSV-1 (IC_{50} = 11.5 μg/mL)	*Plantago ovata* seed husk	(Mukherjee, et al. 2021)
2P	Polysaccharide	Arabinoxylan sulfates	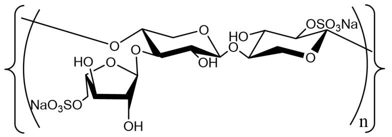	Anti-HSV-1 (IC_{50} = 2.9 μg/mL)	*Plantago ovata* seed husk	(Mukherjee, et al. 2021)
3P		Kappa carrageenan	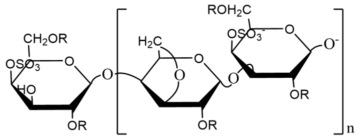	Anti-EV71 (plaque reduction >60%) Anti-SARS-CoV-2 (IC_{50} = 37 ± 20 μg/mL)	Marine red alga *Halymenia durvillei*	(Chiu, et al. 2012) (Schutz, et al. 2021)
